# Genome-wide transcriptomic analysis of a superior biomass-degrading strain of *A. fumigatus* revealed active lignocellulose-degrading genes

**DOI:** 10.1186/s12864-015-1658-2

**Published:** 2015-06-16

**Authors:** Youzhi Miao, Dongyang Liu, Guangqi Li, Pan Li, Yangchun Xu, Qirong Shen, Ruifu Zhang

**Affiliations:** Jiangsu Key Lab and Engineering Center for Solid Organic Waste Utilization, National Engineering Research Center for Organic-based Fertilizers, Nanjing Agricultural University, Nanjing, 210095 P.R. China; Key Laboratory of Microbial Resources Collection and Preservation, Ministry of Agriculture, Institute of Agricultural Resources and Regional Planning, Chinese Academy of Agricultural Sciences, Beijing, 100081 P.R. China

**Keywords:** Plant biomass, Lignocellulose, *Aspergillus fumigatus*, WGS, Transcriptomics

## Abstract

**Background:**

Various saprotrophic microorganisms, especially filamentous fungi, can efficiently degrade lignocellulose that is one of the most abundant natural materials on earth. It consists of complex carbohydrates and aromatic polymers found in the plant cell wall and thus in plant debris. *Aspergillus fumigatus* Z5 was isolated from compost heaps and showed highly efficient plant biomass-degradation capability.

**Results:**

The 29-million base-pair genome of Z5 was sequenced and 9540 protein-coding genes were predicted and annotated. Genome analysis revealed an impressive array of genes encoding cellulases, hemicellulases and pectinases involved in lignocellulosic biomass degradation. Transcriptional responses of *A. fumigatus* Z5 induced by sucrose, oat spelt xylan, Avicel PH-101 and rice straw were compared. There were 444, 1711 and 1386 significantly differently expressed genes in xylan, cellulose and rice straw, respectively, when compared to sucrose as a control condition.

**Conclusions:**

Combined analysis of the genomic and transcriptomic data provides a comprehensive understanding of the responding mechanisms to the most abundant natural polysaccharides in *A. fumigatus*. This study provides a basis for further analysis of genes shown to be highly induced in the presence of polysaccharide substrates and also the information which could prove useful for biomass degradation and heterologous protein expression.

**Electronic supplementary material:**

The online version of this article (doi:10.1186/s12864-015-1658-2) contains supplementary material, which is available to authorized users.

## Background

Plant biomass is the most abundant natural material on earth and the only foreseeable sustainable source of fuels and materials available to humanity [1, 2]. It mainly consists of cellulose, hemicellulose, lignin, pectin and other polymers in ratios that vary between plant species [3]. Cellulose, the main polymeric component of plant biomass [4], pt?>consists of D-glucoside residues linked by beta-1,4-glycosidic bonds to form linear polymeric chains of over 10000 glucose residues. Cellulose usually contains regions that are highly crystalline in nature [5]. The hydrolysis of cellulose and the degradation of the other polysaccharides in plant biomass require many types of enzymes (e.g., endoglucanase, β-glucosidase, cellobiohydrolase, endo-β-1,4-xylanase, β-xylosidase and numerous other associated enzymes) that work in concert [6]. Even when enzymes work synergistically, degradation of recalcitrant lignocellulose is more difficult than degradation of many other natural materials, such as starch [3].

The key step for the utilization of plant biomass (such as agricultural crop residues, grasses, wood and municipal solid waste) is to degrade it into sugars or oligosaccharides by means of various hydrolytic enzymes. The central technological impediment is the general absence of low-cost technology for overcoming the recalcitrance of lignocellulosic biomass, as the costs of cellulases and hemicellulases contribute substantially to the price [7, 8]. Studies aimed at understanding and increasing the efficiency and productivity of plant biomass-degrading enzymes are at the forefront of this research. By combining enzyme engineering methods (such as mutagenesis, DNA shuffling and protein recombination) with effective screening protocols [9], fold-increased activity and thermostability of enzymes have been achieved [10–12]. However, protein engineering strategies are not always effective and may only make relatively small changes to existing enzymes. Consequently, screening of high-efficiency strains and gene resources is critical for improving biomass utilization [9].

Filamentous fungi are widely used for their capacity to produce extracellular proteins in large quantities [13, 14]. As a result of their saprotrophic lifestyle, filamentous fungi can secret a wide range of enzymes into their habitats, enabling them to metabolize a variety of plant polysaccharides. One filamentous fungus, *Neurospora crassa*, has long served as a model fungus [15, 16] and the molecular techniques developed for *N. crassa* studies have also been widely applied to other filamentous fungi [17]. Meanwhile, *Aspergillus* genus species (including *A. niger*, *A. nidulans* and *A. oryzae*) have long been used in the enzymatic industry [18, 19]. Another industrial fungus, *Trichoderma reesei*, was reported to have the ability to produce 100 g of extracellular cellulases per liter [20]. Whole genomes of all of these fungi have been sequenced, which has greatly facilitated the understanding of their highly efficient biomass-degradation mechanisms [21, 22], the design of enzyme cocktails [5, 23], and the engineering of strains for higher protein expression.

As we know, *A. fumigatus* is as versatile in nature as other model fungi. However, there are few detailed studies of its ability to degrade plant biomass, even though some strains of this species are recognized as potent producers of lignocellulolytic and hemicellulolytic enzymes [24, 25]. In our previous study *A. fumigatus* Z5 isolated from compost heaps of plant straw, has been shown to produce highly thermostable lignocellulosic enzyme activities [26]. Many of the glycoside hydrolases are closely related and have redundant (or partially overlapping) functions, which may facilitate the survival of *A. fumigatus* Z5 under different environmental conditions (e.g., variations in pH, temperature, ionic strength) or may be necessary to effectively depolymerize complex carbohydrate polymers. In order to further understand the biomass-degradation mechanism used by this fungus and to facilitate the development of its applications, the genome sequence of *A. fumigatus* Z5 was determined and the transcriptomic profiles induced by sucrose, xylan, cellulose and rice straw were compared. Combined analysis of the genomic and transcriptomic data provides a comprehensive understanding of the responding mechanisms to the most abundant natural polysaccharides in *A. fumigatus*, as well as a roadmap for biomass utilization and the industrial application of gene resources or as a host for protein expression.

## Results

### General genomic features and comparison with several industrial strains

Phylogenetic analysis (Additional file 1) of strain Z5 and other species in the *Aspergillus* section *Fumigati* was based on the β-tubulin, calmodulin and ITS1 and 2 (rRNA) sequences [27], confirming the attribution of Z5 to *A. fumigatus*. The *A. fumigatus* Z5 genome was sequenced using the 454 GS FLX platform [28]. A total of 596 contigs were assembled from the reads. Of the contigs, 523 were longer than 2 kb. The N50 has a length of 97 kb (that is, 50 % of all bases are contained in contigs of at least 97 kb). These contigs were assembled into 24 scaffolds with a total length of 29.4 Mb and an N50 length of 2.29 Mb. The genome was predicted to contain 9540 genes that encode proteins with a length greater than 80 amino acid residues. The genome was composed of 8 chromosomes, as determined by matching scaffolds with the chromosomes of *A. fumigatus* AF293 [29] (Additional file 2). Strain Z5 has 245 unique proteins when compared to *A. fumigatus* AF293 and *N. fischeri* NRRL 181, and of these unique proteins, 218 were annotated as hypothetical proteins. Genome statistics are presented in Table 1. Several Z5 scaffolds matched different chromosomes of *A. fumigatus* AF293, suggesting that duplication of genomic fragments likely occurred in strain Z5 during the evolution of Z5 from the common ancestor.

**Table 1:** Genome features of *A. fumigatus* Z5

Features	Values
Sizes (bp)	29,369,860
Chromosomes	8
G + C content (%)	49.2 %
Protein coding genes	9540
Mean gene length (bp)	1719
Percent coding	55.9 %
Percent genes with introns	82.3 %
Genes of unknown function	21.2 %
Exons	
Mean number per gene	3.28
Mean length (bp)	463.5
G + C content (%)	54.0 %
Introns	
Mean number per gene	2.28
Mean length (bp)	87.3
G + C content (%)	45.5 %
Intergenic regions	
Mean length (bp)	1356
G + C content (%)	44 %
RNA	
tRNA	189

**Fig. 1:**
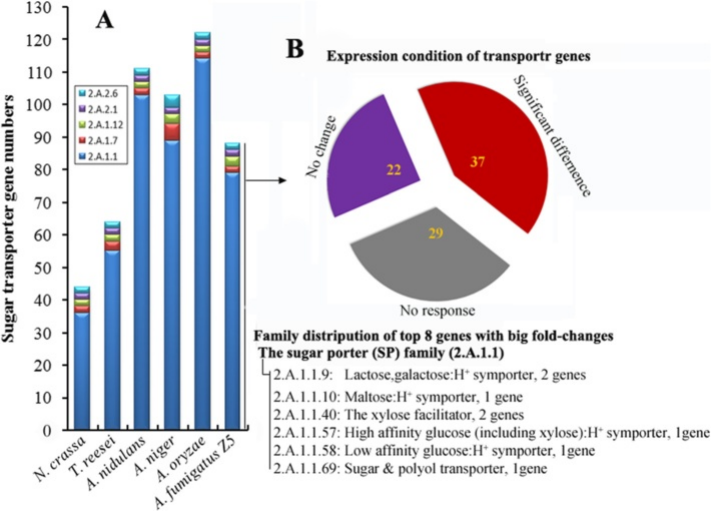
The sugar transporter genes. **a** Comparison of the sugar transporter genes in six filamentous fungi. 2.A.1.1, the sugar porter (SP) family; 2.A.1.7, the fucose:H^+^ symporter (FHS) family; 2.A.1.12, the sialate:H^+^ symporter (SHS) family; 2.A.2, the glycoside-pentoside-hexuronide (GPH):cation symporter family. **b** Expression conditions of the sugar transporter genes in strain Z5. Red indicated differently expressed genes in polysaccharides-induced samples when compared to sucrose control, gray represented low expression genes (RPM < 20) in all samples including sucrose control and purple represented the considerable expressed genes but no differences among 4 treatments. The exact families were given for these 8 genes which expression changed the most

*A. fumigatus* Z5 has 627 significantly different protein-coding genes compared to other 5 well-studied filamentous fungi (Table 2). InterPro [30] identification of conserved domains and families among predicted proteins of *A. fumigatus* Z5 could provide an overview of the expression capability of this filamentous fungus (Additional files 3 and 4). A large expansion in the InterPro category corresponding to the major facilitator superfamily (MFS) in *Aspergillus* genus may reflect their powerful ability to transport small solutes compared with 2 other filamentous fungi, *T. reesei* and *N. crassa*. About the number of sugar transporters in MFS transporters, *Aspergillus* genus has an average of 106 sugar transporters, nearly two folds of those in *T. reesei* and *N. crassa*, and this expansion of Z5 was happened in the sugar porter (SP) family (TC no. 2.A.1.1) which covered 90 % of all the sugar transporters in these fungi (Fig. 1). Consistent with the capacity for efficient lignocellulose degradation, strain Z5, like three other *Aspergillus* species (*A. niger, A. nidulans, A. oryzae*), has a relatively high number of glycoside hydrolase superfamily genes (IPR017853). Compared to *T. reesei* and *N. crassa*, the *Aspergillus* genus has more fungal-specific transcription factors (IPR007219), suggesting that *Aspergillus* species have more complex or redundant regulation mechanisms. *A. fumigatus* Z5 has 81 cytochrome P450 genes (IPR001128), which play important roles in secondary metabolism, while *A. fumigatus* AF293 has only 65 cytochrome P450 genes [31]. In order to identify lignocellulose-degrading proteins, the protein domains encoded by the *A. fumigatus* Z5 genome were compared with the genomes of six other fungi widely used in industry (Additional file 4). The *Aspergillus* strains were found to contain more glycoside hydrolases, pectate lyases (pectin esterases) and tannases (feruloyl esterases) than when compared to *T. reesei* and *N. crassa*.

**Table 2:** Specific proteins in six filamentous fungi

Species	Specific proteins	Total proteins
*A. fumigatus* Z5	627	9540
*A. niger*	3664	14097
*A. nidulans*	1006	10680
*A. oryzae*	1158	12030
*N. crassa*	2645	9907
*T. reesei*	1374	9143

**Fig. 2:**
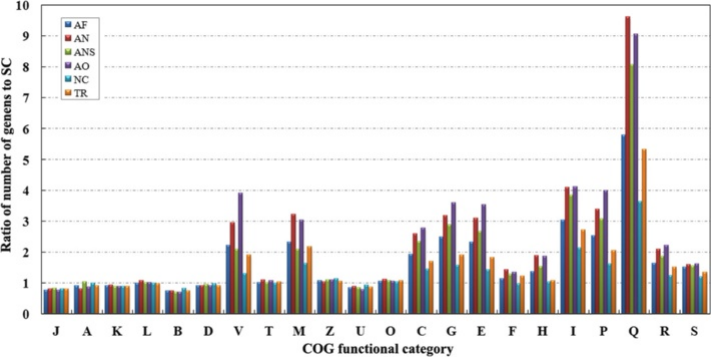
Comparison of relative gene numbers in each COG. The ratios of gene counts in *A. fumigatus* Z5 (AF), *A. niger* (AN), *A. nidulans* (ANS), *A. oryzae* (AO), *N. crassa* (NC) and *T. reesei* (TR) to gene counts in *S. cerevisiae* (SC) in each COG category were calculated. COGs with a gene number ≤5 for each species (Y, N and W) are not displayed to avoid misinterpretation resulted from potentially low reliability. J, Translation, ribosomal structure and biogenesis; A, RNA processing and modification; K, Transcription; L, Replication, recombination and repair; B, Chromatin structure and dynamics; D, Cell cycle control, cell division, chromosome partitioning; Y, Nuclear structure; V, Defense mechanisms; T, Signal transduction mechanisms; M, Cell wall/membrane/envelope biogenesis; N, Cell motility; Z, Cytoskeleton; W, Extracellular structures; U, Intracellular trafficking, secretion, and vesicular transport; O, Posttranslational modification, protein turnover, chaperones; C, Energy production and conversion; G, Carbohydrate transport and metabolism; E, Amino acid transport and metabolism; F, Nucleotide transport and metabolism; H, Coenzyme transport and metabolism;I, Lipid transport and metabolism; P, Inorganic ion transport and metabolism; Q, Secondary metabolites biosynthesis, transport and catabolism; R, General function prediction only; S, Function unknown

Using the cluster of orthologous groups (COG) classification database [32], all available proteins from six filamentous fungi and a strain of *Saccharomyces cerevisiae* were clustered into 25 COG functional categories (Fig. 2; see also Additional file 5). There were no differences in any of the information storage and processing categories (J to B) or in most of the cellular processes and signaling categories (D to O other than V and M), even though there were large differences in genome sizes. Most of the gene family expansions in filamentous fungi, when compared to *Saccharomyces cerevisiae*, were found in gene families predicted to have roles in defense mechanisms (V), extracellular structures (M) and metabolism (C to E, I to Q). Genes in these expanded categories were more abundant in the *Aspergillus* genus than in *N. crassa* or *T. reesei*.

### Carbohydrate-active enzymes in *A. fumigatus* Z5

Carbohydrate-active enzymes (CAZymes) are categorized into different classes (glycoside hydrolases, glycosyltransferases, polysaccharide lyases, carbohydrate esterases, auxiliary activities and carbohydrate-binding modules) in the CAZy database (http://www.cazy.org) [33]. CAZymes cleave, build and rearrange oligo- and polysaccharides. These functions play important roles in fungi such as *A. fumigatus* and are vital for optimizing biomass degradation by these species [5]. Given the importance of this protein family for biomass utilization, a detailed examination of the CAZome of *A. fumigatus* Z5 was performed.

**Fig. 3:**
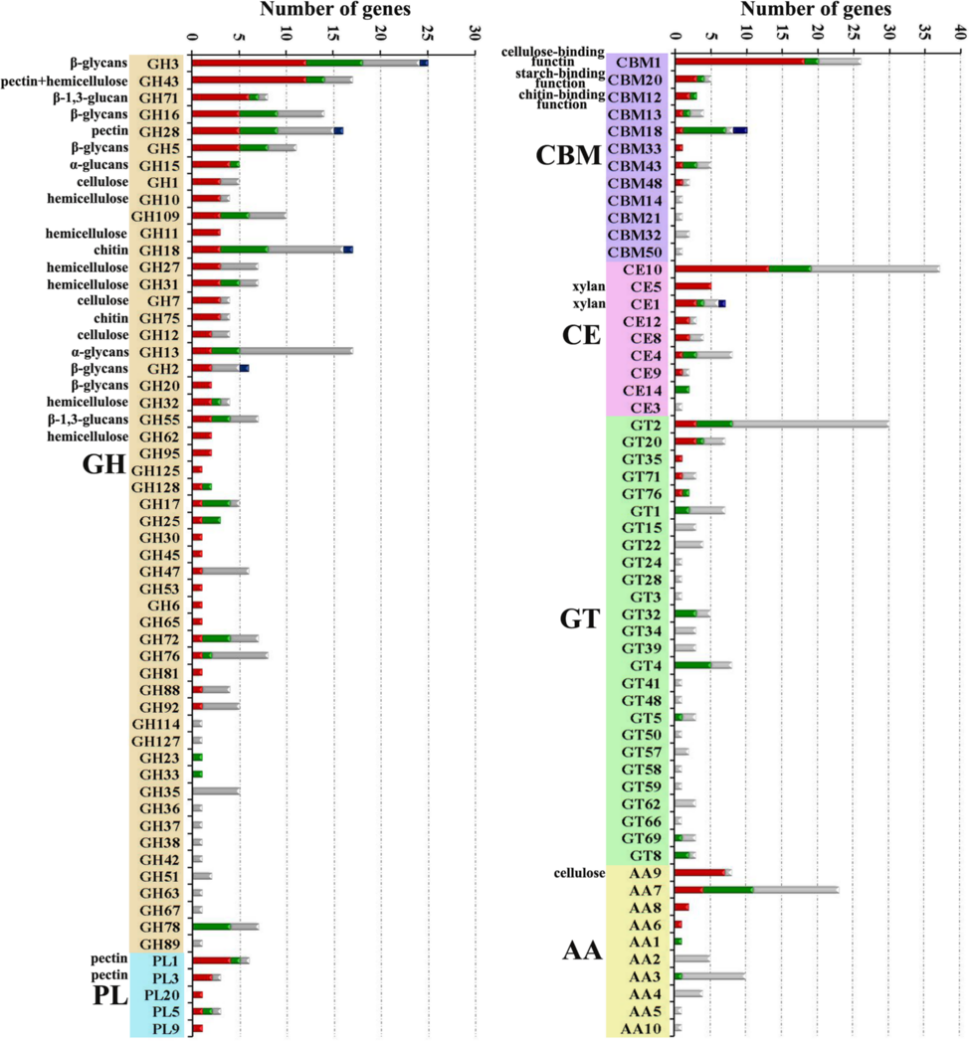
The distribution of each CAZyme family in *A. fumigatus* Z5. The bars depict the numbers of CAZyme genes in each family. The red portion of the bar indicates the number of genes in each CAZyme family that are induced when strain Z5 is incubated with the polysaccharides xylan, cellulose or rice straw as the sole carbon source, the green bars are down-regulated gene numbers in three polysaccharides when compared to sucrose control, the grey bars are no response genes and the dark blue bars represent the genes which showed the opposite expression trends in three polysaccharides when compared to sucrose control. GH, glycoside hydrolases; CBM, carbohydrate binding module; CE, carbohydrate esterase; GT, glycosyltransferases; PL, polysaccharide lyase; AA, auxiliary activities. Known substrates or activities of some CAZyme families are given

The genome of *A. fumigatus* Z5, which efficiently degrades plant polysaccharides, encodes approximately 529 putative carbohydrate-active enzymes (280 glycoside hydrolases, 98 glycosyltransferases, 14 polysaccharide lyases, 69 carbohydrate esterases, 58 auxiliary activities and 61 carbohydrate-binding modules), which were divided into at least 115 distinct families (Additional file 6). Compared to several available *A. fumigatus* and *N. fisheri* genomes in the *Aspergillus* section *Fumigati* (Table 3), no significant differences were found in the distribution of CAZyme classes between our strain Z5 and other three *A. fumigatus*, and 92.3 % CAZyme proteins in *N. fisheri* genome showed homogenous with those of *A. fumigatus*. In fact, *Aspergillus* genus has the leading position in the number of CAZyme genes among the 103 publicly available fungal genomes [34]. Figure 3 depicts the distribution of CAZyme genes by family and the responses of gene families to the induction by extracellular polysaccharides. Of the glycoside hydrolase genes, 39.2 % had significantly higher expression levels (q-value ≤ 0.0001 and |the ratio of the RPM values| ≥ 4) after polysaccharide induction than after sucrose induction (as discussed below). Most of the glycoside hydrolases with significantly increased expression were responsible for the degradation of cellulose, hemicellulose and pectin (Fig. 3). Under the same conditions, the carbohydrate-binding module, carbohydrate esterase, auxiliary activities and polysaccharide lyase families also had a relative high percentage of induced genes (45.9, 39.1, 24.1 and 64.3 % respectively). However, glycosyltransferases appeared to fulfill a secondary or supportive role in polysaccharides degradation because only a very small percentage of genes were induced, 9.1 %.

**Table 3:** CAZyme gene numbers in *A. fumigatus* and *N. fischeri*

Species	CAZyme proteins	GH			GT			CE			PL			CBM			AA		
		N.	H.	F.	N.	H.	F.	N.	H.	F.	N.	H.	F.	N.	H.	F.	N.	H.	F.
*A. fumigatus* Z5	528	272	99 %	53	98	98 %	26	69	99 %	9	14	100 %	5	61	97 %	12	55	100 %	10
*A. fumigatus* AF293	528	269	100 %	53	109	99 %	27	75	100 %	9	15	100 %	6	62	97 %	11	57	100 %	9
*A. fumigatus* var. RP-2014	498	257	100 %	53	99	100 %	24	68	100 %	9	15	100 %	6	54	96 %	11	50	100 %	9
*A. fumigatus* A1163	532	270	100 %	53	106	99 %	25	73	99 %	9	15	100 %	6	60	97 %	10	58	100 %	9
*N. fischeri* NRRL 181	595	304	97 %	54	103	106	25	87	81 %	8	17	88 %	7	78	90 %	12	66	91 %	10

Enzymes: GH, glycoside hydrolase; GT, glycosyltransferase; CE, carbohydrate esterase; PL, polysaccharide lyase; CBM, carbohydrate-binding module; AA, auxiliary activities. Genomes: *N. fischeri* NRRL 181 (GenBank: AAKE00000000.3); *A. fumigatus* AF293 (GenBank: AAHF00000000.1); *A. fumigatus* var. RP-2014 (GenBank: JHOI00000000.1); *A. fumigatus* A1163 (GenBank: ABDB00000000.1). N.: total numbers of each classes; H.: percentage of homologs compared among these five fungi; F.: family numbers of each classes

**Fig. 4:**
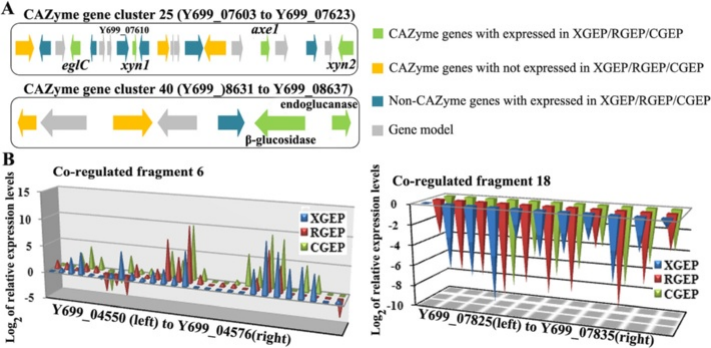
CAZyme gene clusters and co-regulated fragments. **a** CAZyme gene clusters: cluster 25 contained a beta-1,3-endoglucanase gene (*eglC*) and an acetyl xylan esterase gene (*axe1*), as well as 2 endo-1,4-beta-xylanase genes (*xyn1* and *xyn2*). Y699_7610 encodes an extracellular phytase. In CAZyme gene cluster 40, beta-glucosidase and endoglucanase are co-induced. **b** Co-regulated fragments: fragment 6 is up-regulated. It includes the CAZyme gene cluster 6 (from Y699_04554 to Y699_04576), in which Y699_04562 encoded a beta-glucosidase, Y699_04563 encoded a MFS sugar transporter, Y699_04570 encoded a xylosidase/arabinosidase and Y699_04574 encoded a MFS alpha-glucoside transporter. Fragment 18 includes the secondary metabolite biosynthetic gene cluster 23 in which genes are down-regulated in all three treatments compared to sucrose. Y699_07834 encoded a polyketide synthase

**Fig. 5:**
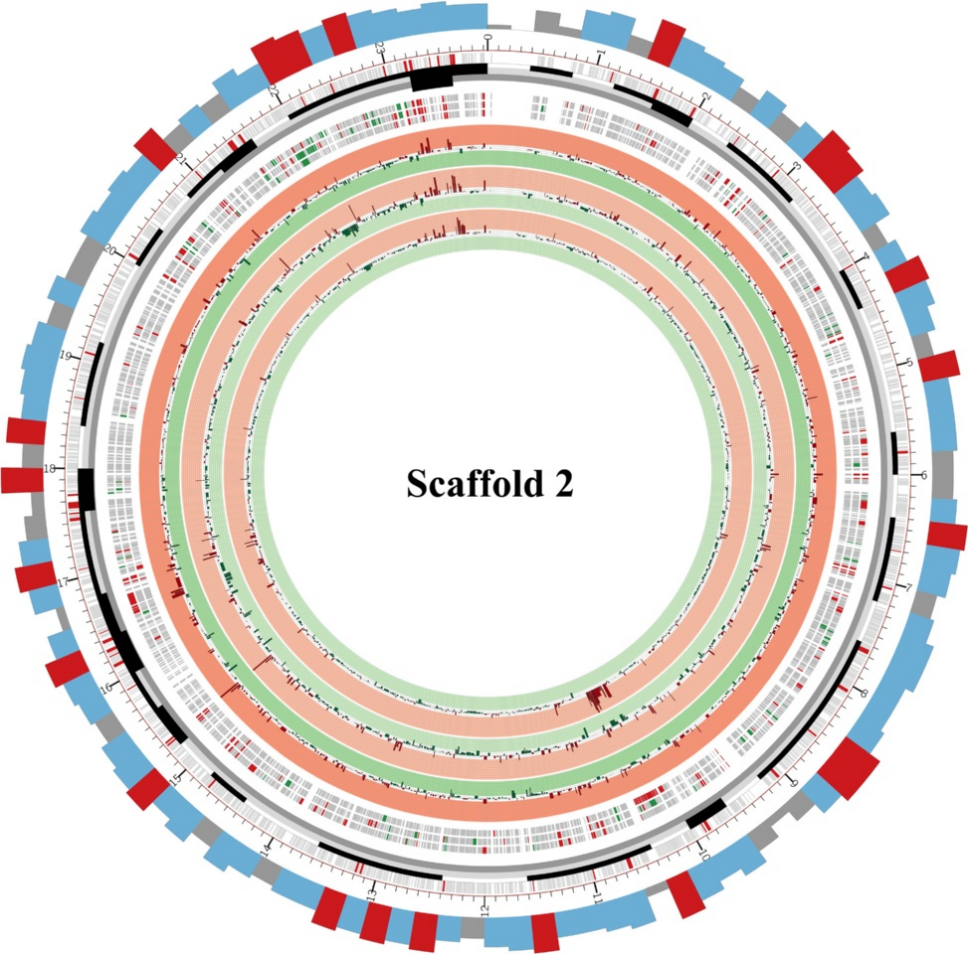
Map of scaffold 2. The first (outermost) circle represents the gene density of scaffold 2. The red indicates gene number higher than 8 in that 20 kb fragment, the blue indicates gene number from 6 to 8 in that 20 kb fragment, and the grey indicates gene number below 6 in that 20 kb fragment. The second circle represents scaffold size, and the smallest unit represents 10 kb. The third circle represents all genes in scaffold 2. CAZyme genes are indicated in red. The discrete black boxes describe the density of CAZyme genes. The next three circles indicate the genes which expression differs significantly in xylan, rice straw or cellulose, from outside to inside, respectively, when compared to sucrose as a control condition. Up-regulated genes are red, and down-regulated genes are green. The innermost three circles are gene expression graphs in cellulose, rice straw and xylan relatively to sucrose, from outside to inside, respectively. A red background indicates log_2_ (ratio of RPM values) ≥ 2, and a green background indicates log_2_ (ratio of RPM values) ≤ -2). Scaffold 2 has a CAZyme gene cluster that spans the bases 2300-2370 kb in which all genes were up-regulated relatively to sucrose in the 3 treatments

Plant biomass contains cellulose, hemicellulose, pectin and other natural materials such as lignin, the degradation of which requires many enzymes working synergistically. Cellulose degradation requires endoglucanases (EGs), cellobiohydrolases (CBHs) and β-glucosidases. EGs catalyze the hydrolysis of cellulose at random positions in less crystalline regions, and CBHs act processively on the non-reducing ends of the chains to release the disaccharide cellobiose, which is cleaved by β-glucosidases to yield glucose [12]. A thorough inspection of the Z5 genome revealed at least 25 EGs, 3 CBHs and at least 15 β-glucosidases (Additional file 7). EGs in Z5 belong to six glycoside hydrolase families (GH5, GH7, GH12, GH16, GH45, AA9), with seven EGs in these families having CBM1 domains (Y699_02044, Y699_02871, Y699_03689, Y699_4295, Y699_05430, Y699_07518 and Y699_08692, respectively). Two CBHs from strain Z5 belong to the family GH7 (CBH1) and another belongs to the family GH6 (CBH2). Z5 has four β-glucosidases in family GH1 and 11 in GH3. The cellulase genes of *A. fumigatus* Z5 are not closely adjacent in the genome except for an endoglucanase (Y699_04295) and a cellobiohydrolase (Y699_04296). In addition to the enzymes responsible for the degradation of cellulose, numerous other plant cell wall polysaccharide-degrading enzymes were also predicted in the *A. fumigatus* Z5 genome, including hemicellulases and pectinases, which have catalytic activities for degradation of hemicelluloses and pectin, respectively (Additional file 7). Analysis of the CAZyme gene locations in the Z5 genome revealed that CAZyme genes were nonrandomly distributed within the genome. Of the 529 CAZyme genes, 209 were located in one of 44 discrete regions, which ranged from 3.6 kb to 175 kb in length (roughly 1.8 Mb in total) (Fig. 4 and Additional file 8). Each region contained two to 13 CAZyme genes. Most of the CAZyme genes in the clusters were not from the same subfamily, which indicated that gene relocation rather than duplication was responsible for the formation of the CAZyme gene clusters. Approximately 59.3 % (124) of the CAZyme genes in the clusters encoded glycoside hydrolases. The fact that 46.3 % of the glycoside hydrolase genes and 26.5 % of the glycosyltransferase genes were located in these clusters indicated that the majority of the CAZymes were involved in polysaccharide degradation. Furthermore, of the genes that were differentially expressed in treatment conditions (induction by xylan, cellulose or rice straw) when compared to the sucrose as a control condition, most were located in or around the CAZyme-rich regions (Fig. 5 and Additional file 9).

### Protein secretion of *A. fumigatus* Z5

In contrast to other microorganisms, filamentous fungi can secret large amounts of proteins into the external environment. Strain Z5 is also an extraordinarily efficient producer of extracellular enzymes [25, 26], which makes the analysis of the protein secretion system particular interesting. Here, we compared some components of secretory pathway among strain Z5 and 5 other industrially relevant filamentous fungi extensively used for protein secretion (*A. niger*, *A. nidulans*, *A. oryzae*, *N. crassa* and *T. reesei*). Translocation from the cytoplasm to the endoplasmic reticulum (ER) occurs in these fungi through established signal recognition particle (SRP)-dependent and SRP-independent pathways [35], which are very important steps for heterologous gene expression. Orthologs of most *S. cerevisiae* proteins in these two pathways were found in strain Z5 and 5 other fungi except for a yeast signal recognition and docking protein, Srp21p [36]. All these filamentous fungi have a gene encoding protein disulphide isomerase (PDI) (ortholog of yeast Pdi1p) and 3 other PDI-related genes. The chaperone BiP, a member of the heat shock 70 protein family, is more conserved (similarity > 90 %) than other components of secretory pathways in these fungi, reflecting the central role of BiP in translocation into the ER, protein folding and regulation response [37, 38]. Multiple enzymes are required for N-glycosylation of glycoproteins, which involves binding of a preformed oligosaccharide (Glc3Man9GlcNAc2) to asparagine side chains of an immature protein. All six fungi have at least one copy of the key enzymes for this process (glucosidase I and II, calnexin, calreticulin and UDP-glucose:glycoprotein glucosyltransferase). which indicates that they may have a common folding and quality control system for glycoproteins. The unfolded protein response (UPR) signaling pathway in these fungi differs somewhat from that of yeast. In mammalian cells, the PERK (double-stranded RNA-activated protein kinase-like endoplasmic reticulum kinase) machinery can attenuate protein translation by phosphorylating the alpha subunit of eukaryotic translation initiation factor 2 (eIF2α) in the early phase of ER stress [39]. PERK is then inhibited by binding to the HSP40 cochaperone p58, the expression of which is induced by IRE1/XBP1 of the UPR pathway. The ortholog of p58 was found in the genomes of these fungi but not in yeast, and the ortholog of PERK was not found or had low similarity. Homologs of most known ERAD components were found in all these filamentous fungi, despite an apparent lack of orthologs and little sequence similarity to yeast ERAD components, as reported for the *A. niger* genome [36]. Some counterparts of the mammalian GTPase proteins (Rab2, Rab4, Arf6 and Arf10), which are involved in membrane fusion and vesicle budding in diverse cellular locations, were found in all these fungi, whereas they were absent in yeast [40].

**Fig. 6:**
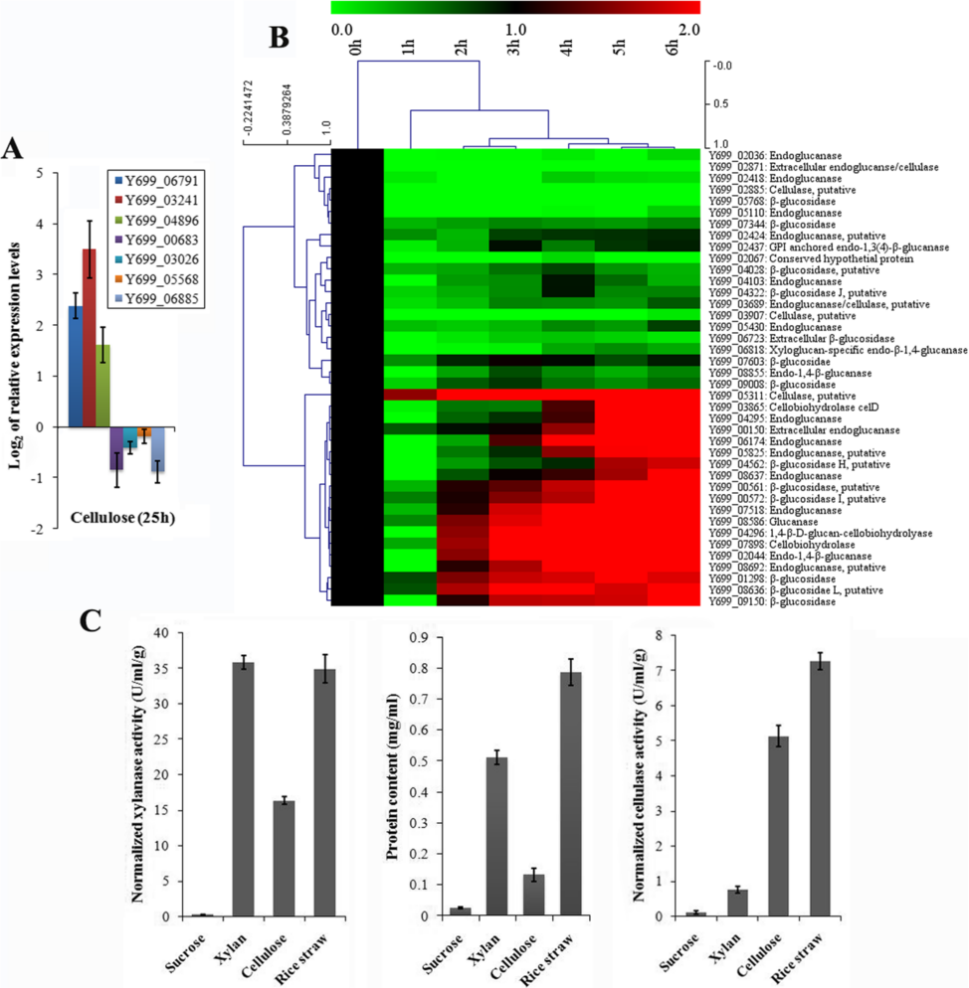
The validation of transcriptomic data. **a** Seven genes in protein synthesis were validated by qRT-PCR after been induced by cellulose for 25 h. **b** All predicted cellulase genes were investigated under the induction of cellulose at time points of 0 h, 1 h, 2 h, 3 h, 4 h, 5 h and 6 h after sucrose replaced by cellulose. The color bar indicates the range of lg of relative expression levels in the heat map figures. To distinguish induced and non-induced genes, the values at 0 h for each gene were set to 1. **c** After been induced for 25 h, extracellular protein concentration and normalized enzyme activities (U/ml/g of fungal dry weight) were measured for three polysaccharide samples and sucrose control

In order to get insight into the secretory pathway of *A. fumigatus* Z5, transcriptional profiles of genes in the secretory pathway induced by different carbon sources were analyzed (Additional file 10). Most genes had higher expression levels when grown on sucrose than on the other three carbon sources (xylan, cellulose and rice straw as the sole carbon source). This is interesting because the samples with more extracellular proteins have low gene transcription in protein synthesis. qRT-PCR results of several selected genes verified the transcriptomic data (Fig. 6a). These results indicated that the polysaccharides-induced samples actually had a relative low stress of protein synthesis when compared to sucrose control. Sucrose, as a favored carbon source, could promote the growth of strain Z5 much faster than other polysaccharides.

### Transcriptional responses of *A. fumigatus* Z5 to different carbon sources

**Fig. 7:**
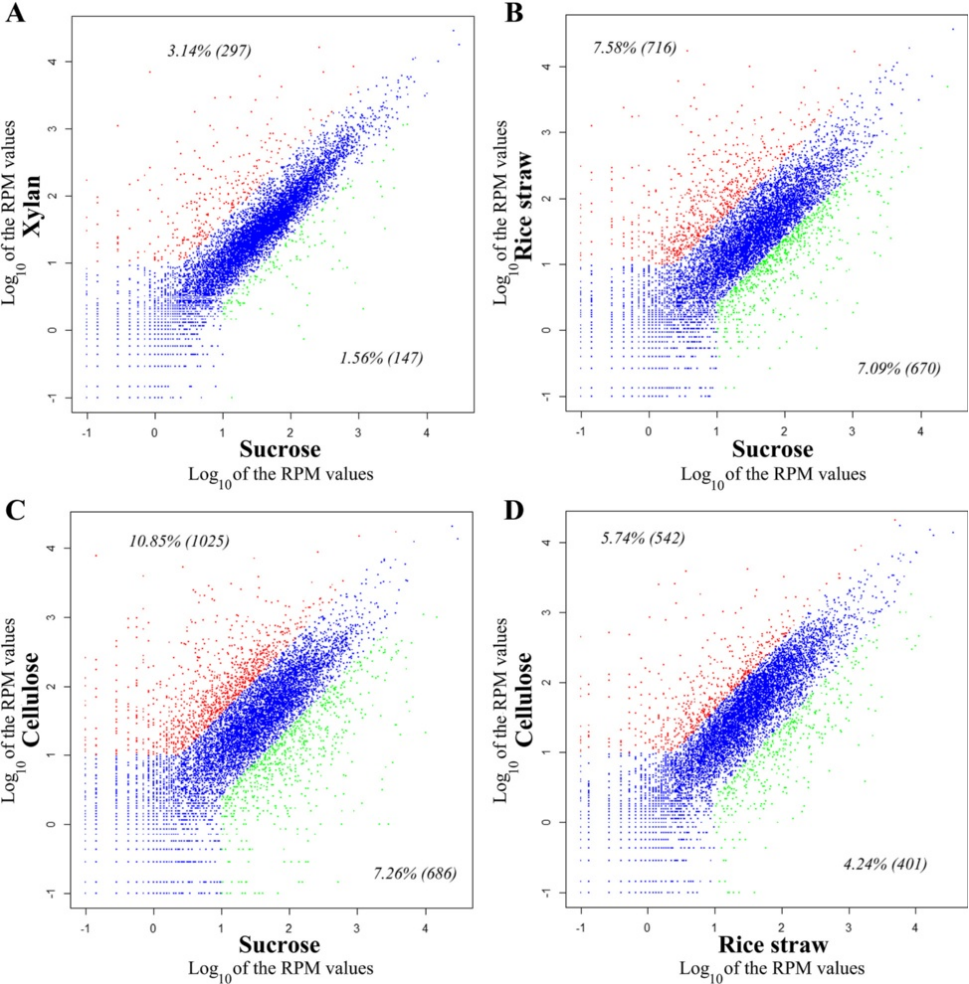
Comparison of different gene expression patterns induced by different substrates. Gene expression patterns induced by xylan, cellulose and rice straw are compared to gene expression induced by sucrose (**a**, **b** and **c**, respectively), and gene expression patterns induced by rice straw are compared to those induced by cellulose (**d**). Every point represents a gene with expression levels (RPM values) that vary in 2 transcriptomes. Blue points indicate genes that have no significant differences in 2 transcriptomes, red points indicate up-regulated genes and green points indicate down-regulated genes. The number of genes significantly up- or down-regulated in each transcriptome is reported in parentheses

**Fig. 8:**
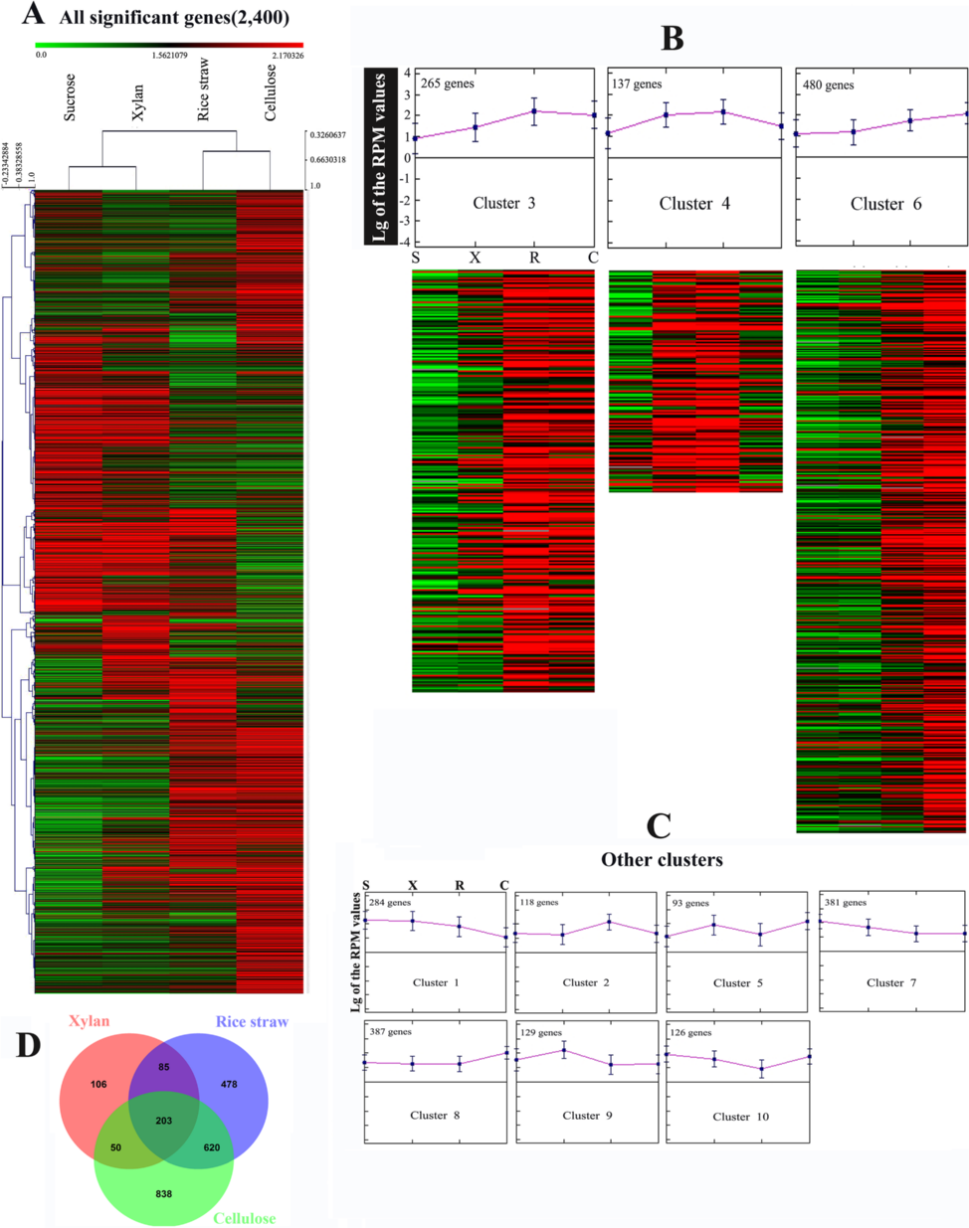
Gene expression profiles for substrate-responsive genes. The color bar indicates the range of expression levels in the heat map figures **a** Genes with significantly different expression are depicted (see Methods). **b** Three clusters are depicted with heat map figures and centroid graphs. In cluster 3 and cluster 4, genes are expressed at a higher level in all 3 treatments (xylan, cellulose and rice straw) compared to sucrose. In cluster 6 genes are only highly expressed when growing in cellulose and rice straw. **c** The other seven clusters, Vertical bars on graphs represent the data range, and the points in the middle represent the average. **d** Venn diagram obtained from the comparison of the significantly differently expressed genes induced by different polysaccharides when compared to sucrose control

**Fig. 9:**
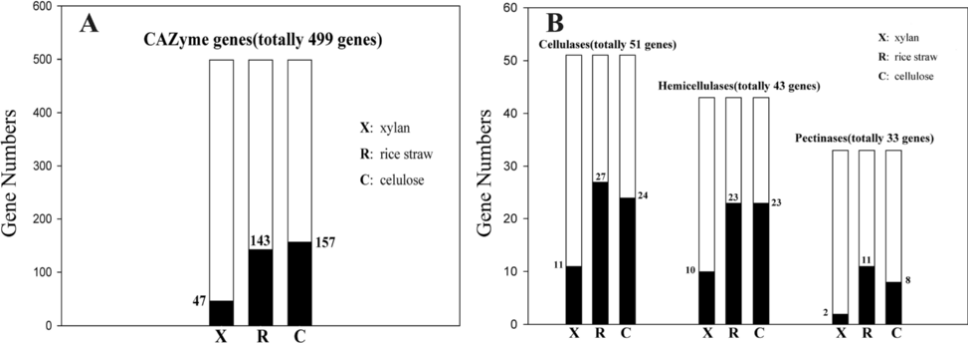
The conditions of degrading-enzyme gene expression. The whole histogram includes all genes in that group, and black indicates the genes that were significantly different in that treatment compared to sucrose. **a** CAZyme gene numbers with significantly different expression levels in xylan, rice straw and cellulose when compared to sucrose control. **b** Hydrolase genes with significantly different expression levels in xylan, rice straw and cellulose compared to sucrose

Genome analysis showed the potential of a wide spectrum of polysaccharide hydrolytic enzymes produced by *A. fumigatus* Z5. In order to determine which hydrolytic enzyme-encoding genes were induced by different substrates (sucrose, xylan, cellulose and rice straw), the transcriptional profiles of *A. fumigatus* Z5 under these conditions were determined. Xylan, as the major component of hemicellulose, was used as a sole carbon source to induce the expression of hemicellulase genes, while cellulose was used as the inducer of cellulases. Rice straw, which contains various polysaccharide components, was chosen as a carbon source to study the diversity of the degradation enzymes. *A. fumigatus* Z5 was grown on 2 % sucrose (used as control), and the mycelia were transferred to 1 % (w/v) xylan, cellulose or rice straw as the sole carbon source for 16 h. Gene expression patterns revealed that comparable genes were differentially expressed in each transcriptome, and many had similar patterns (Figs. 7 and 8). The xylan-induced gene expression pattern (XGEP) is relatively similar to the sucrose-induced gene expression pattern (SGEP), which may be due to the high efficiency of xylan degradation (data not shown) and xylose-induced carbon catabolite repression [41, 42]. However, the cellulose-induced gene expression pattern (CGEP) and the rice straw-induced gene expression pattern (RGEP) differ significantly from the SGEP (Fig. 7b, c). The main aim of this study was to focus on genes that have significantly different expression among the 4 different treatments. Four hundred and two genes were identified in cluster 3 and cluster 4, which showed higher expression levels in the XGEP, CGEP and RGEP than in the SGEP. These two clusters contained nearly all of the polysaccharides-degrading genes, including 14 cellulases (7 EGs: Y699_00150, Y699_02424, Y699_05430, Y699_06174, Y699_07518, Y699_08637 and Y699_08692; 2 CBHs: Y699_03865 and Y699_07898; 3 β-glucosidases: Y699_00561, Y699_01298 and Y699_08636), nine xylanases (5 endo-1,4-beta-xylanases: Y699_04481, Y699_06333, Y699_07611, Y699_07623 and Y699_09486; 4 β-xylosidases: Y699_04570, Y699_04662, Y699_05610 and Y699_07880), 2 alpha-L-arabinofuranosidases and 2 acetyl xylan esterases. Seven other cellulases (3 EGs: Y699_02044, Y699_04295 and Y699_05825; 4 β-glucosidases: Y699_00572, Y699_04562, Y699_06723 and Y699_09150) were found in cluster 6 and were expressed at higher levels in the CGEP and RGEP than in the SGEP and XGEP. Together, these results show that both cellulose and xylan can induce the expression of cellulase and xylanase genes, but xylan induces fewer cellulase genes (Fig. 9). Three cellobiose dehydrogenase (*cdh*) genes (Y699_02120, Y699_04685 and Y699_05507) were detected in the *A. fumigatus* Z5 genome. Consistent with a recent report that cellobiose dehydrogenase and a copper-dependent polysaccharide monooxygenase can enhance cellulose degradation [43], we found that the *cdh* gene (Y699_02120) was induced by the substrates cellulose and rice straw. Gene Y699_04685 in cluster 2 only had a high expression level in RGEP compared to the three other patterns, suggested that this cellobiose dehydrogenase may not functional in the degradation of cellulose and xylan but may be useful for degradation of other components of plant biomass, such as pectin or other aromatic substrates. Interestingly, two endoglucanase genes (Y699_02418 and Y699_05110) were also located in cluster 2, which indicated that these two genes may not functional in the degradation of cellulose due to their low expressional levels in the CGEP.

To validate the transcriptomic results, all the predicted cellulase genes were investigated by qRT-PCR under the induction of cellulose, which confirmed the transcriptomic results (Fig. 6b). Additionally, extracellular protein contents and total xylanase and cellulase activities of the 4 treatments (sucrose, cellulose, xylan and rice straw) provide further evidences to the transcriptomic data (Fig. 6c).

The MFS includes 82 protein families, 6 of which are known to transport sugars (the sugar porter (SP) family, the oligosaccharide:H^+^ symporter (OHS) family, the fucose:H^+^ symporter (FHS) family, the sialate:H^+^ symporter (SHS) family, the polyol permease (PP) family and the sugar efflux transporter (SET) family), in addition, the glycoside-pentoside-hexuronide (GPH):cation symporter family is also responsible for sugars transportation [44]. Totally, strain Z5 has 88 sugar transporter genes distributed among SP, FHS, SHS and GPH families (SP family includes 79 genes), 37 genes were differently expressed in polysaccharides-induced samples when compared to sucrose control, the 8 genes which expression changed the most were predicted to transport glucose, xylose, lactose and maltose. Three (Y699_03783, Y699_04557 and Y699_04571) and six sugar transporter genes (Y699_01300, Y699_02331, Y699_03701, Y699_07512, Y699_09366 and Y699_09270) were only induced by xylan and cellulose, respectively. Gene Y699_09366 was up-regulated about 200 folds by both cellulose and rice straw, which is the homolog of a lactose permease gene (Gene ID: 3450) essential for cellulase induction in *T. reesei* v2.0 [45]. All the homologs of the 37 genes could be detected in other 5 filamentous fungi (*A. niger*, *A. nidulans*, *A. oryzae*, *N. crassa* and *T. reesei*) even though *N. crassa* only has 44 sugar transporter genes, which indicated that filamentous fungi shared common mechanisms of sugar transportation.

**Fig. 10:**
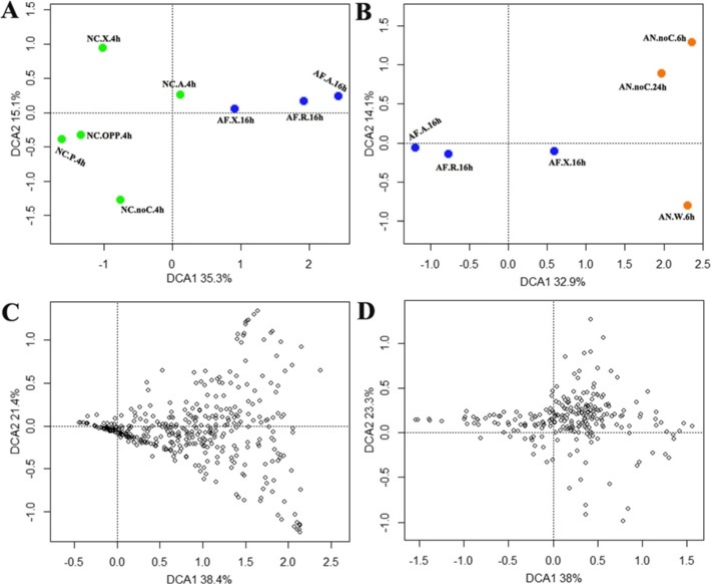
Comparison of different fungal expression profiles induced by polysaccharides. Expression profiles induced by polysaccharides were compared between strain Z5 and other two filamentous fungi, *N. crassa* and *A. niger*, by the method of detrended correspondence analysis. The percentages of the first two principal components explaining the expressional variations were given in axis tags. **a** Expression profiles of 5557 homologous genes were compared between strain Z5 and *N. crassa*. AF.X.16h, AF.A.16h and AF.R.16h: induction by xylan, avicel and rice straw respectively for 16 h in *A. fumigatus* Z5. NC.X.4h, NC.A.4h, NC.P.4h, NC.OPP.4h and NC.noC.4h: induction by xylan, avicel, pectin, orange peel power and no carbon respectively for 4 h in *N. crassa*. **b** Expression profiles of 7246 homologous genes were compared between strain Z5 and *A. niger*. AN.W.6h and AN.noC.6h: induction by wheat straw and no carbon respectively for 6 h in *A. niger*. AN.noC.24h: induction by no carbon for 24 h in *A. niger*. **c** 392 homologous CAZyme genes were compared between strain Z5 and *A. niger*, which were induced by polysaccharides and no carbon (shown up). **d** 241 homologous CAZyme genes were compared between strain Z5 and *N. crassa*

Strain Z5 has 7246 and 5569 homologous genes with *A. niger* and *N. crassa* respectively. Comparison of the transcriptomic data of Z5, *A. niger* (GEO DataSets series: GSE57315) and *N. crassa* (GEO DataSets series: GSE42692) under the induction of different polysaccharides (glucose served as control for *A. niger*, sucrose for stain Z5 and *N. crassa*) found that avicel- and rice straw-induced transcriptomic profiles of strain Z5 were very similar, and orange peel power (OPP)- and pectin-induced transcriptomic profiles of *N. crassa* were similar (Fig. 10a), however, the responses of the three fungi to the same polysaccharide were significantly different, xylan-induced transcriptomic profile of strain Z5 was similar to avicel-induced gene transcriptomic profile of *N. crassa*. The 7246 homologs of strain Z5 and *A. niger* include 391 CAZymes, some of which had the similar responses to the same polysaccharide (Fig. 10c), this trend was also observed between *N. crassa* and strain Z5 (Fig. 10d). Comparison of the straw induced transcriptomic profiles of different strains, transcription of 1386 genes were significantly changed by the induction of rice straw in strain Z5 when compared to sucrose, however, in *A. niger* and *T. reesei*, only 514 and 529 genes’ transcription were changed (defined by the same standard), respectively, by the induction of wheat straw [22, 46]. Among these differently expressed genes, only 154/94 homologs were found between strain Z5 and *A. niger*/*T. reesei* (Additional file 11), and most of these homologs were cellulases, xylanases, sugar transporters and hypothetical proteins. In general, filamentous fungi showed the diverse performances to plant biomass in the whole transcriptome level, but they shared a fair amount of similarity on plant biomass degradation.

### GO enrichment analysis

Analysis of expression patterns in different treatments indicated that 444, 1386 and 1711 genes were up-regulated or down-regulated in the XGEP, RGEP and CGEP, respectively, in comparison to the SGEP (Fig. 8d). A GO term enrichment analysis of the three polysaccharide-induced gene expression patterns compared to the SGEP was performed with the topGO R package (Additional file 12) [47]. XGEP vs. SGEP analysis showed enrichment of transmembrane transport (P = 1.1e-05), the D-xylose metabolic process (P = 1.3e-04), the xylan catabolic process (P = 7.5e-04) and the polysaccharide binding category (P = 9.3e-04). Comparison of RGEP with SGEP indicated enrichment of transmembrane transport (P = 1.4e-06), the cellulose catabolic process (P = 6.0e-05), the xylan catabolic process (P = 8.4e-06), the pectin catabolic process (P = 5.5e-05) and the polysaccharide binding category (P = 5.8e-12). The comparison of CGEP with SGEP revealed enrichment of carbohydrate transport (P = 7.5e-04), the cellulose catabolic process (P = 7.6e-05), the hemicellulose metabolic process (P = 7.5e-04), the polysaccharide binding category (P = 6.2e-10) and ribosome biogenesis (P = 1.4e-18). Together, these results show that *A. fumigatus* Z5 could adjust its gene expression and metabolic pathways by using different substrates as a carbon source. When xylan was supplied, Z5 had a higher expression level of xylanase genes and carbohydrate transporter genes, which could degrade xylan into xylobiose or xylose and then transport them into the cell. Xylobiose and/or xylose could be utilized to produce energy or synthesize other materials in the D-xylose metabolic process. Degradation enzymes, transporters and polysaccharide binding activity were also needed during the degradation of cellulose and rice straw. Intriguingly, CGEP showed enrichment of ribosome biogenesis, indicating that *A. fumigatus* Z5 needs more ribosomes for protein synthesis. The protein products might be secreted into the external environment and contribute to the difficult process of crystalline cellulose degradation.

Analysis of the transcriptome profiles across all scaffolds (Fig. 5 and Additional file 9) indicated that the expression levels of most genes were stable, while significant changes occurred in and around CAZyme-rich regions. A total of 468 genes were found in the 35 fragments; they ranged from 8 kb to 112 kb in length, and all were up-regulated or down-regulated in at least 1 of the 3 transcriptomes (Fig. 4 and Additional file 13). Among these fragments, eight segments corresponding to eight different CAZyme gene clusters were found, and five of them were up-regulated in all three transcriptomes compared to SGEP, while the others were down-regulated. These results suggested that the genes in and around the eight CAZyme gene clusters might be closely related to utilization of different carbon sources. Additionally, 11 of the 30 secondary metabolite biosynthetic gene clusters (Additional file 14) in the genome were located or partly located in the co-regulated fragments. Of those 11, five were up-regulated, and the remainders were down-regulated. Therefore, the secondary metabolism pathways might be stimulated in *A. fumigatus* Z5 when the complex organic substances exist in the environment, enabling the strain Z5 to fend off competition for nutrients and survive in complex environmental conditions.

## Discussion

Filamentous fungi efficiently degrade plant biomass and secret substantial amounts of extracellular enzymes into their substrates. Several model filamentous fungi, including *N. crassa*, *T. reesei*, *A. niger*, *A. oryzae* and *A. nidulans*, are widely used in industry and have been the subjects of numerous studies [16, 31, 36, 40, 48]. Unlike the three model species in the *Aspergillus* genus that have a long history of safe use for industrial enzyme production, *A. fumigatus* strains are opportunistic pathogens that may cause invasive aspergillosis in immunocompromised patients [29, 49], although strains in this species are also effective at biomass degradation. *A. fumigatus* Z5 was isolated from crop compost heaps [26], suggesting that the strain is widely distributed in the environment and contributes to the degradation of plant biomass. There have been some studies related to polysaccharide degradation in *Aspergillus fumigatus* [24, 26, 50, 51]. This study on strain Z5 showed that it has an abundant distribution of CAZyme genes in genome and can produce large amount of extracellular enzymes, which make it possible to be a producer of enzymes to degrade plant biomass. Also, as a fast growing filamentous fungus at a high temperature of 50 °C, strain Z5 could easily be a host for heterologous protein expression by the mature molecular technologies in *A. fumigatus*. Even though, strain Z5 was not as good as some well-studied fungi, such as *A. niger* MGG029 with many genomic modifications [49], but it shared a similar potential and results of the present study enable valuable resources for further explorations. The safety of this strain should be considered by knocking out some virulence-related genes, such as the genes for gliotoxin, fumagillin, aflatoxin and ribotoxin [52].

In general, filamentous fungi cannot absorb polysaccharides directly into their cells to use as sources of carbon and energy. Instead, they secrete many types of enzymes to depolymerize polysaccharides into monomeric carbohydrates that are efficiently taken up and metabolized. In eukaryotic cells, protein secretion involves ER-associated translation, folding and glycosylation. Proteins are then moved into the Golgi apparatus or other compartments via vesicles to form the mature proteins. Finally, within secretory vesicles, the mature proteins are transported to the cell membrane and secreted into the environment [53]. Guillemette *et al.* [54] reported transcriptional responses of *A. niger* when exposed to secretion stress (treatment with tunicamycin, treatment with DTT or forced secretion of a heterologous protein). In total, 11 genes in the secretory pathway were induced by the three ER-stress conditions. However, among the homologous counterparts of these 11 genes in *A. fumigatus* Z5, seven were down-regulated in all three treatments (xylan, cellulose and rice straw) when compared to the sucrose as a control condition (Additional file 10). The same trend was observed among the important chaperones and foldases (*bipA*, *pdiA* and *clxA*), which are involved in protein folding in the ER, with the exception of a 1.3-fold increase in bipA observed on cellulose. Unfolded protein response (UPR) and ER-associated degradation (ERAD) were also not induced by the three polysaccharides. These results indicate that there was no ER stress in these conditions compared to sucrose, even though more extracellular proteins were secreted [53]. Two genes involved in glycosylation, the α-1, 2-mannosidase gene (Y699_04911) and the α-glucosidase gene (Y699_09020), exhibited 1.5–22.3-fold and 3–76.4-fold increases, respectively, in the three treatments compared to sucrose control. It has been suggested that plant biomass-degrading enzymes are highly glycosylated [55]. Our study indicates that when polysaccharides are the sole substrate for growth, high levels of extracellular enzymes do not cause ER stress. Indeed, it suggested that if the balance between growth and protein secretion of fungi is managed well, the secretory pathway can be fully explored, and fold-increased production of extracellular proteins can be achieved.

XlnR is a xylanolytic transcriptional activator specific to fungi [56] that effects a wide range of target genes, e.g., genes encoding xylan-degrading enzymes or the enzymes in the D-xylose metabolic pathway [57, 58] and even genes encoding cellobiohydrolases and endocellulases [59]. In this study, the XlnR gene was significantly up-regulated by cellulose and rice straw but not by xylan. Andersen *et al.* [60] reported that in the fermentations of glucose and xylose, 22 conserved genes were differentially transcribed (21 up-regulated and one down-regulated) in three *Aspergillus* species (*A. niger*, *A. oryzae* and *A. nidulans*) when xylose, rather than glucose, was the substrate. Enzymes involved in D-xylose metabolism were among the 22 genes. In the present study, homologs of the 21 up-regulated genes were mostly transcribed at high levels in the three treatments compared with sucrose control (Additional file 15). However, the results indicated that D-xylose metabolism was not activated by XlnR because its transcriptional level was similar in xylan and sucrose, even though the D-xylose metabolic pathway was significantly induced in xylan. The down-regulated gene identified by Andersen *et al.* encoded a MFS monosaccharide transporter, which was reported to have a higher affinity for glucose than xylose in their results. The expression level of the homolog gene decreased considerably in the three polysaccharides treatments of this study, suggesting that either the cellulose and rice straw treatments did not have enough glucose to induce the transporter gene or cellulose was transported into cells mainly as a form of cellobiose rather than glucose [61]. Another interesting finding in the 22 genes is that a sugar transporter (Y699_05888) had a extremely high expression level in xylan and rice straw (81.9-folds and 64.6-folds, respectively) but only a 4.7-folds enhanced in cellulose, which revealed that this sugar transporter should be a specific transporter of the hydrolyzing products from xylan in all four *Aspergillus* species (qRT-PCR validated that gene Y699_05888 was up-regulated 2.8 ± 0.08 and 27.2 ± 0.52 folds by cellulose and xylan, respectively).

Transcriptomic data showed that genes encoding hydrolytic enzymes involved in plant cell wall degradation were repressed during growth on sucrose. Carbon catabolite repression (CCR) is an important mechanism to repress the production of polysaccharide-degrading enzymes during growth on easily metabolized carbon sources. In most fungal species, CCR is mediated by a zinc-finger transcription factor CreA [62–64], which is a repressor for genes encoding polysaccharide-degrading enzymes. In this study, we expected to have low CreA expression levels in the 3 polysaccharides treatments when compared to sucrose treatment. However, the *cre*A gene was transcribed at high levels (RPM = 4714.2 in rice straw and RPM = 3366.3 in cellulose), with 7.7- and 5.5-fold increases in rice straw and cellulose compared to sucrose, respectively. Reverse transcription quantitative PCR (RT-qPCR) experiments under the same conditions as the transcriptomic study confirmed these observations (data not shown). These results suggest that CreA has functions beyond those of a transcription repressor and merits further research.

Our previous research identified many plant biomass-degrading enzymes secreted by Z5 in the presence of different carbon sources (i.e., glucose, cellulose and rice straw) [25]. The expression of those enzymes in the present study is consistent with the findings of the previous study. Additionally, xylanase genes were induced more by cellulose and rice straw than by xylan; xylan was not the best inducer of its own hydrolytic enzymes. We observed that cultivation broth containing xylan became transparent, while culture filtrates with avicel or rice straw still had unused substrates after 16 h of incubation, indicating that pure xylan was depolymerized faster than avicel and rice straw; our previous work showed that Z5 has very strong xylanase activities [25]. We conclude that the simple carbon source, xylose, which is released by xylan degradation, inhibited the persistent high expression of many xylanases. This may also account for the smaller transcriptional differences between the xylan treatment and sucrose control than those observed when either cellulose or rice straw treatments are compared to the control.

## Conclusions

This study performed transcriptional analysis on an *A. fumigatus* strain grown on xylan, cellulose and rice straw. It comprehensively described natural polysaccharide saccharification by *A. fumigatus* hydrolases and the regulation of gene expression that occurs during this process. Having identified enzymes actively involved in biomass degradation, we foresee the potential to increase enzyme activities in industrial contexts. This would first require the heterologous expression of the enzymes at high levels and optimization of their incorporation into enzymatic cocktails. The genome and transcriptome data of this study are also valuable for future studies of aspergillosis.

## Methods

### Genome assembly, scaffolding, and gene prediction

The genome of *A. fumigatus* strain Z5 was shotgun sequenced using a Roche 454 GS FLX system at the Chinese National Human Genome Center (Zhangjiang Hi-tech Park, Shanghai, China). It produced 837 Mb of sequence data (29.9 × coverage) with an average read length of 358 bp. Using the Newbler software (v2.3) within the Roche 454 suite package [65], these reads were assembled into 596 contigs with a total size of 29.4 Mb. For sequence scaffolding, Solexa Mate Pair reads (insert size 3 ~ 5 kb) were used to establish the genome scaffolds. A total of 38,248,825 mate-pair reads contributed to improving sequence quality and constructing scaffolds. A simple greedy algorithm was used to optimize the order of the contigs and provided a feasible heuristic solution for scaffolds construction [66]. By mapping the reads to contigs, 557 contigs were then linked, generating 24 genome scaffolds with their length not smaller than 1 kb. For gene prediction, Genemark (v2.3 a, http://topaz.gatech.edu/GeneMark/) and Augustus (v2.5, http://bioinf.uni-greifswald.de/augustus/) were used to predict genes for the Z5 genome, and the genome of *A. fumigatus* AF293 was used as a reference. The predicted genes were annotated using public databases (NCBI nr, Uniprot and KEGG) with BLAST, and the predicted genes with the best supporting annotation results were selected as the gene models. As a result, a total of 9540 gene models were described in the genome. Transfer RNAs (tRNAs) were predicted with tRNAscan-SE [67] (http://lowelab.ucsc.edu/tRNAscan-SE/). Secreted proteins were identified by SignaIP 4.0 analysis [68] (http://www.cbs.dtu.dk/services/SignalP/).

### Strains and culture conditions

Strain Z5 was grown on potato dextrose agar (PDA) plates for conidia production. Conidia were harvested by washing the plate with 10 ml sterile ddH_2_O. After removing the mycelia, the conidia were resuspended, and the concentration was adjusted to 1 × 10^7^ conidia/ml. Basic culture medium (BCM) was composed of the following: 0.25 g/L yeast extract, 1 g/L MnSO_4_ · H_2_O, 0.5 g/L urea, 0.5 g/L (NH_4_)_2_SO_4_, 0.5 g/L MgSO_4_ · 7H_2_O, 7.5 mg/L FeSO_4_ · 7H_2_O, 2.5 mg/L MnSO_4_ · H_2_O, 3.6 mg/L ZnSO_4_ · 7H_2_O, 3.7 mg/L CoCl_2_ · 6H_2_O, 0.5 g/L CaCl_2_ and a predetermined concentration of carbon source according to our experimental conditions [69]. 1 × 10^7^ conidia/L were inoculated into liquid BCM with sucrose as the carbon source (BCM-S) and cultured at 45 °C for 24 h. For cultivation in a medium containing xylan from oat spelts (Sigma), Avicel PH-101 (Sigma-Aldrich) or rice straw as a carbon source, the mycelia grown in the BCM-S were exhaustively washed with sterile distilled water and then transferred into yeast-free BCM and containing 1 % oat spelts xylan, 1 % Avicel PH-101 (a source of cellulose), or 1 % rice straw as a carbon source for 16 h. Mycelia were harvested by filtration through one layer of gauze, washed thoroughly with sterile water and quickly frozen in liquid nitrogen for further RNA extraction. The reason for sucrose used to be control is that sucrose is a favored carbon source to polysaccharides, and it will not induce any lignocellulolytic genes in filamentous fungi. For BCM with rice straw, the rice straw obtained from the farmland was exhaustively washed with deionized water for several days until reducing sugars were not detected by dinitrosalicylic acid (DNS). The rice straw was then dried completely at 40 °C, made into rice straw powder using a grinder and stored at room temperature.

### Enzyme assays

Extracellular proteins were precipitated by 80 % ammonium sulfate and redissolved in sterile distilled water. The protein concentration was determined with a Micro BCA protein assay kit (Beyotime, China) using bovine serum albumin as a standard. Cellulase and xylanase activities were measured by 3, 5-dinitrosalicylic acid (DNS) method according to Liu *et al.* [25]. One unit of enzyme activity was defined as the amount of enzyme required to release 1 μmol of reducing sugars from the substrate in 1 min.

### RNA extraction and qRT-PCR experiments

The frozen mycelia were disrupted by grinding in liquid nitrogen, and total RNA was extracted using the RNeasy Plant Mini Kit (Qiagen) combined with the RNase-Free DNase set (Qiagen). The total RNA (approximately 2 μg) extracted from each treatment was fractionated on a 1.2 % agarose gel, stained with ethidium bromide and visualized with UV light to check RNA integrity. The total RNA samples were then quantified using a NanoDrop 2000 spectrophotometer (Thermo, USA). For qRT-PCR analysis, cDNA were synthesized from the total RNA in different induction time using a PrimeScript™ RT-PCR Kit (TAKARA, DaLian, China) according to the manufacturer’s instructions. All quantitative PCR runs were performed in triplicate on a 7500 Fast Real-Time PCR System (Applied Biosystems) using SYBR® Premix Ex Taq™ Kit (Tli RNaseH Plus) (TAKARA, DaLian, China). An actin-encoding gene and a histone-encoding gene (GenBank Accession No: Y699_04988 and Y699_07225, respectively) were chosen together to be reference genes. The primer sequences are given in Additional file 16.

### Transcriptome sequencing and gene mapping

Messenger RNA was purified from the total RNA with a Micropoly(A)PuristTM mRNA purification kit (Ambion, America). Double-stranded cDNA was synthesized according to Ng’s full-length cDNA synthesis protocol [70] with some modifications. GsuI-oligo dT was used as the primer for first-strand cDNA synthesis from 10 μg of mRNA, using 1,000 units of Superscript II reverse transcriptase (Invitrogen, America). The diol group of the CAP structure of mRNA was then oxidized by NaIO_4_ (Sigma, America) and linked to biotin hydrazide. Dynal M280 streptavidin Dynabeads (Invitrogen, America) were used to select biotinylated RNA/cDNA. The first-strand cDNA was then released by alkaline hydrolysis. Next, adaptors were ligated to the 5’-end of the first-strand cDNA by DNA ligase (TaKaRa, Dalian, China), and the second-strand cDNA was synthesized through primer extension using Ex Taq polymerase (TaKaRa, Dalian, China). Last, the polyA tail and 5’ adaptors were removed by GsuI digestion. The synthesized cDNA was fractioned ultrasonically into 300-800 bp fragments and purified using Ampure beads (Agencourt, America). The prepared cDNAs were transformed into libraries with the TruSeq DNA Sample Prep Kit Set A (Illumina, America), clonally amplified using the TruSeq PE Cluster Kit (Illumina, America) and sequenced with Illumina equipment according to the manufacturer's instructions. Clean reads (those in which at least half of the bases, excluding N, had a quality score above 5) were retained and mapped onto the genome or annotated genes of strain Z5. Reads were collected from the different samples for each gene, and read counts were transformed into RPM values (Reads Per Kilo bases per Million reads) [71]. Using the MA-plot-based method with Random Sampling model in the DEGseq program package [72], the differences in expression levels between samples were calculated for each individual gene. Differences were determined to be significant if FDR scores were ≤ 0.001.

### Gene expression analysis

The genes with significantly different transcriptional levels in all 4 transcriptomes need to have RPM (Reads Per Kilo bases per Million reads) scores above 10 in all transcriptomes as well as the coefficient of variation of the RPM values in all transcriptomes of not less than 0.6. The results were organized and visualized using Pearson correlations and hierarchical clustering with the average linkage clustering method to view the whole data set. K-means cluster analysis was used to group the genes into 10 clusters with the TIGR multiexperiment viewer (MeV, http://www.tigr.org/software). For the purposes of GO terms enrichment analysis, genes that differed significantly between treatment (XGEP, CGEP or RGEP) and control (SGEP) were those that had RPM ≥ 10, q-value ≤ 0.0001 and |log_2_ of the ratio of the RPM values| ≥ 2. GO terms annotation was accomplished by Blast2GO software (http://www.blast2go.com). Genes that differed significantly between treatment and control were analyzed and visualized to see the results of GO term enrichment using the classic algorithm and Fisher’s exact test in the Bioconductor topGO package (http://www.bioconductor.org/packages/2.12/bioc/html/topGO.html). Detrended correspondence analysis (DCA) was conducted using the vegan package of R [73]. The transcriptome sequencing data and screening results are provided in Additional file 17.

### Protein classification

Whole genome protein families were classified by InterproScan analysis (http://www.ebi.ac.uk/interpro/). The number of genes for each COG category was determined using a BlastP search against the COG data set. The highest scoring hit to COG category for each protein was retained. Using the same way, the sugar transporter family proteins were found out by blasting to Transporter Classification Database (TCDB, http://www.tcdb.org/). CAZyme proteins were classified with the CAZYmes Analysis Toolkit [74] (CAT, http://mothra.ornl.gov/cgi-bin/cat.cgi). Annotation based on sequence similarity and annotation based on association rules between CAZy families and pfam domains against the CAZy database (http://www.cazy.org/) were integrated for CAZyme protein predictions.

### Gene cluster identification

The gene families were collected by visual inspection using CIRCOS software [75] (http://www.circos.ca). For CAZyme gene clusters, a cluster is defined as a region containing a statistically higher proportion of a particular gene family, and the cluster must begin and end with a gene from the family studied. We then calculated the probability of these CAZyme gene clusters based on a hypergeometric distribution, and all 44 clusters had a P value < 0.01. To analyze fungal clusters of secondary metabolism, the genome annotation data were coordinated and analyzed with the program SMURF (http://www.jcvi.org/smurf/index.php). The nucleotide fragments in which genes were nearly co-regulated (up- or down-) were also collected by visual inspection and compared to the clusters of secondary metabolism by Blastp.

### Accession number

This Whole Genome Shotgun project has been deposited at DDBJ/EMBL/GenBank under the accession AZZA00000000. The version described in this paper is version AZZA01000000. The transcriptomes data discussed in this publication have been deposited in NCBI’s Gene Expression Omnibus [76] and are accessible through GEO Series accession number GSE55086 (http://www.ncbi.nlm.nih.gov/geo/query/acc.cgi?acc=GSE55086).

## Additional files

Additional file 1:
**Phylogenetic analysis of strain Z5 in** ***Aspergillus*** **section** ***Fumigati*** **.** Phylogenetic analysis of strain Z5 was performed in Aspergillus section *Fumigati* based on the β-tubulin, calmodulin and ITS sequences. Additional file 2:
**Scaffolds matching.** All Z5 scaffolds were matched into the chromosomes of *A. fumigatus* AF293. Additional file 3:
**Comparison of protein domains in six filamentous fungi.** The numbers of each protein domain identified by InterPro were compared within *A. fumigatus*, *A. nidulans*, *A. niger*, *A. oryzae*, *T. reesei* and *N. crassa*. Additional file 4:
**Plant biomass-related protein families in** ***A. fumigatus*** **Z5.** Plant biomass-related protein families were found out in the six filamentous fungi and *S. cerevisiae*. Additional file 5:
**Comparison of gene numbers for each COG category.** The predicted numbers of each COG category for seven fungi including *A. fumigatus* Z5 were listed. Additional file 6:
**CAZyme genes.** All the predicted CAZyme genes in *A. fumigatus* Z5 were listed. Additional file 7:
**Plant biomass-degrading enzymes in** ***A. fumigatus*** **Z5.** Cellulase genes, hemicellulase genes and pectinase genes were found out from the genome of Z5, and the properties (protein family, signal peptide, secretome signal and homologs in other fungi) of these enzymes were also investigated. Additional file 8:
**CAZyme gene clusters in** ***A. fumigatus*** **Z5.** 44 CAZyme gene clusters were found in Z5 genome, and p-values < 0.01. Additional file 9:
**Maps of scaffolds.** The map of scaffold 2 was shown by Fig. 4 with the detailed descriptions, and this file contained the maps for the remained scaffolds. Additional file 10:
**Differential expression of secretory pathway genes.** This file shown the expression values of genes in secretory pathway in *A. fumigatus* Z5 when induced by different carbon source. Additional file 11:
**Homologs among the rice straw-induced strain Z5 and wheat straw-induced** ***A. niger*** **and** ***T. reesei*** **.** This file listed the homologs of genes that were differently expressed in rice straw-induced Z5, wheat straw-induced *A. niger* and *T. reesei*. Additional file 12:
**GO enrichment analysis. 444, 1386 and 1711 significantly differently expressed genes (xylan to sucrose, rice straw to sucrose and cellulose to sucrose, respectively) were used to perform GO enrichment analysis.** The results were shown in this file. Additional file 13:
**Co-regulated fragments in Z5 genome.** 35 co-regulated fragments consisting of continuous genes in genome were found and listed in this file. Additional file 14:
**Secondary metabolite.** 30 secondary metabolite biosynthetic gene clusters and related PKS genes NPRS genes in Z5 genome were shown in this file. Additional file 15:
**Co-regulated genes in** ***Aspergillus*** **genus when grown on xylose.** 23 co-regulated genes in *Aspergillus* genus identified by Andersen *et al.* when grown on xylose rather than glucose were also conserved in transcriptome data in this study. Additional file 16:
**The primers for qRT-PCR experiments.** The primers of 53 genes were listed in this file. Additional file 17:
**Transcriptome screening results.** This file contained the selected genes for the analysis of Pearson correlations and hierarchical clustering and GO enrichment.
