# Supplementary material for: Genome-wide transcriptomic analysis of a superior biomass-degrading strain of A. fumigatus revealed active lignocellulose-degrading genes
Source: BMC Genomics. 2015 Jun 16;16(1):459. doi: 10.1186/s12864-015-1658-2 (PMC4469458; doi:10.1186/s12864-015-1658-2)
Supplement: Additional file 1: — Phylogenetic analysis of strain Z5 in Aspergillus section Fumigati . Phylogenetic analysis of strain Z5 was performed in Aspergillus section Fumigati based on the β-tubulin, calmodulin and ITS sequences. [file 12864_2015_1658_MOESM1_ESM.docx]

**Additional file 1: Phylogenetic analysis of strain Z5 in *Aspergillus* section *Fumigati*.**

*N. aurata* NRRL 4378

*N. stramenia* NRRL 4652

*N. assulata* KACC 41691

*A. unilateralis* NRRL 577

*N. galapagensis* IBT 16763

*N. multiplicata* CBS 646.95

*N. tatenoi* NRRL 4584

*N. nishimurae* IFM 54133

*A. turcosus* KACC 41955

*N. glabra* NRRL 3434

*N. fennelliae* NRRL 5535

*N. denticulata* CBS 652.73

*N. hiratsukae* KACC 41688

*N. udagawae* CBM FA 0702

*N. aureola* NRRL 2244

*A. viridinutans* NRRL 4365

*N. spinosa* NRRL 5034

*N. laciniosa* KACC 41657

*A. novofumigatus* IBT 16806

*A. fumigatiaffinis* IBT 12703

*N. coreana* KACC 41659

*A. fumisynnematus* IFM 42277

*A. lentulus* NRRL 35552

*N. fischeri* NRRL 4161

**strain Z5 in this study**

*A. fumigatus* NRRL 163

*N. spathulata* NRRL 20550

*N. pseudofischeri* NRRL 20748

*N. quadricincta* NRRL 2154

*A. brevipes* NRRL 2439

*A. duricaulis* NRRL 4021

99

99

99

99

98

98

53

97

82

92

67

82

54

74

50

0.01

**Fig. 1.** Neighbour-joining tree based on β-tubulin sequence data of *Aspergillus* section *Fumigati*. Numbers above branches are bootstrap values. Only values above 50% are indicated.

*A. viridinutans* NRRL 4365

*N. aureola* NRRL 2244

*N. udagawae* CBM FA 0702

*N. nishimurae* IFM 54133

*N. assulata* KACC 41691

*A. turcosus* KACC 41955

*A. unilateralis* NRRL 577

*N. multiplicata* CBS 646.95

*N. hiratsukae* KACC 41688

*N. spathulata* NRRL 20550

*N. glabra* NRRL 3434

*N. galapagensis* IBT 16763

*N. stramenia* NRRL 4652

*N. aurata* NRRL 4379

*N. tatenoi* NRRL 4584

*A. brevipes* NRRL 2439

*A. duricaulis* NRRL 4021

*N. quadricincta* NRRL 2154

*N. pseudofischeri* NRRL 20748

*N. denticulata* CBS 652.73

*N. fennelliae* NRRL 5535

*N. spinosa* NRRL 5034

*N. coreana* KACC 41659

*N. laciniosa* KACC 41657

*A. fumisynnematus* IFM 42277

*A. lentulus* NRRL 35552

*A. novofumigatus* IBT 16806

*A. fumigatiaffinis* IBT 12703

*A. fumigatus* NRRL 163

**strain Z5 in this study**

*N. fischeri* NRRL 4161

99

99

99

99

91

99

90

89

72

87

97

89

63

57

93

85

63

73

86

90

0.01

**Fig. 2.** Neighbour-joining tree based on calmodulin sequence data of *Aspergillus* section *Fumigati*. Numbers above branches are bootstrap values. Only values above 50% are indicated.

**Fig. 3.** Neighbour-joining tree based on ITS sequence data of *Aspergillus* section *Fumigati*. Numbers above branches are bootstrap values. Only values above 50% are indicated.

*N. coreana* KACC 41659

*A. novofumigatus* KACC 41934

*A. fumigatiaffinis* UOA/HCPF 9455/9518/9540

*N. laciniosa* KACC 41657

*A. fumisynnematus* IFM 42277

*N. spinosa* NRRL 5034

**strain Z5 in this study**

*A. fumigatus* NRRL 163

*N. fischeri* NRRL 4161

*A. lentulus* NRRL 35552

*A. viridinutans* NRRL 4365

*N. aureola* NRRL 2244

*N. udagawae* KACC 41156

*N. nishimurae* IFM 54133

*N. multiplicata* IFM 53594

*N. quadricincta* NRRL 2154

*A. duricaulis* NRRL 4021

*N. assulata* IBT 27911

*N. aurata* NRRL 4379

*N. stramenia* NRRL 4652

*N. fennelliae* NRRL 5535

*A. turcosus* KACC 41955

*N. glabra* NRRL 3434

*A. unilateralis* NRRL 577

*A. brevipes* NRRL 2439

*N. tateno* NRRL 4584

*N. spathulata* NRRL 20550

*N. pseudofischeri* NRRL 20748

96

56

67

54

50

62

63

50

52

0.002
