# Supplementary material for: Genome-wide transcriptomic analysis of a superior biomass-degrading strain of A. fumigatus revealed active lignocellulose-degrading genes
Source: BMC Genomics. 2015 Jun 16;16(1):459. doi: 10.1186/s12864-015-1658-2 (PMC4469458; doi:10.1186/s12864-015-1658-2)

Additional file 2: all scaffolds matched to the chromosomes of *Aspergillus fumigatus* AF293

a: **scaffolds matched to chromosome 1 of af293** (y-axis: all scaffolds, x-axis: chromosome 1)

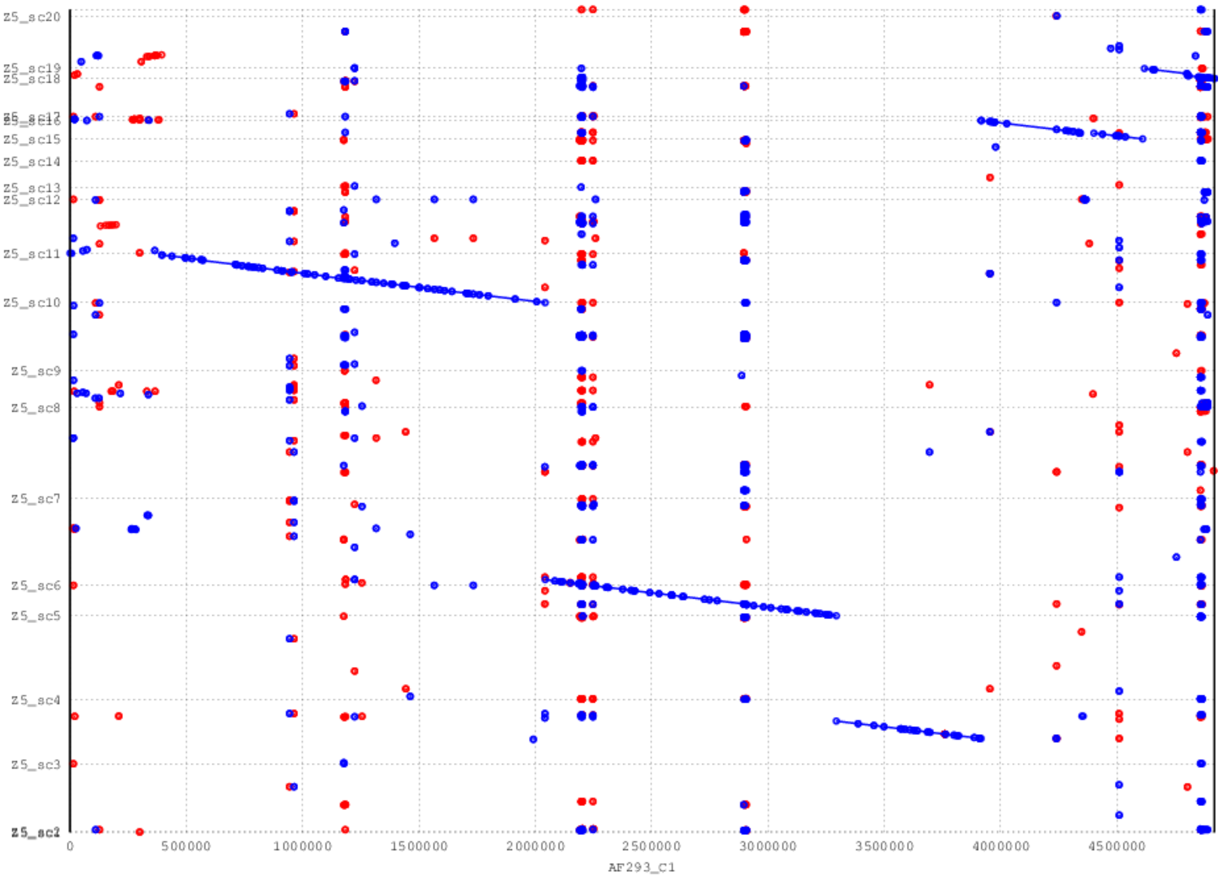

b: **scaffolds matched to chromosome 2 of af293** (y-axis: all scaffolds, x-axis: chromosome 2)

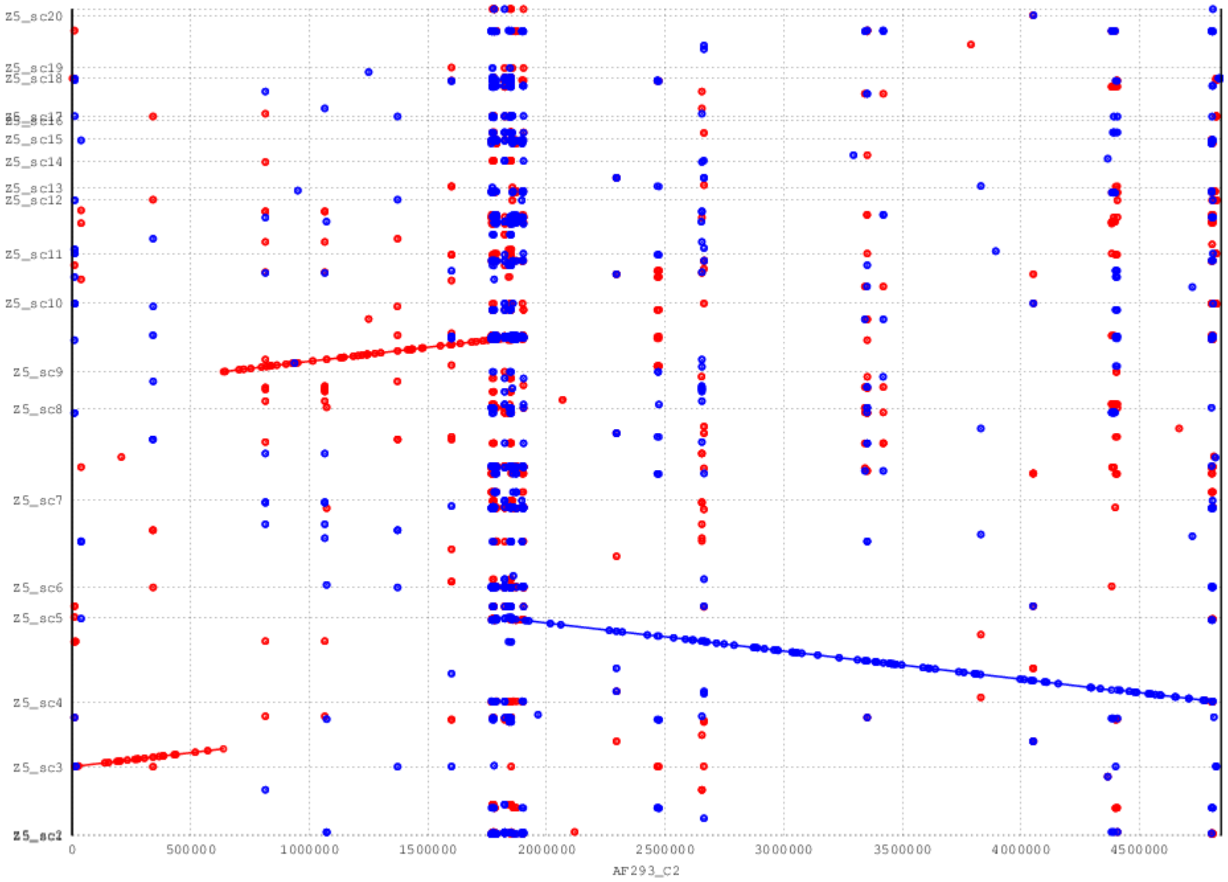

c: scaffolds matched to chromosome 3 of af293 (y-axis: all scaffolds, x-axis: chromosome 3)

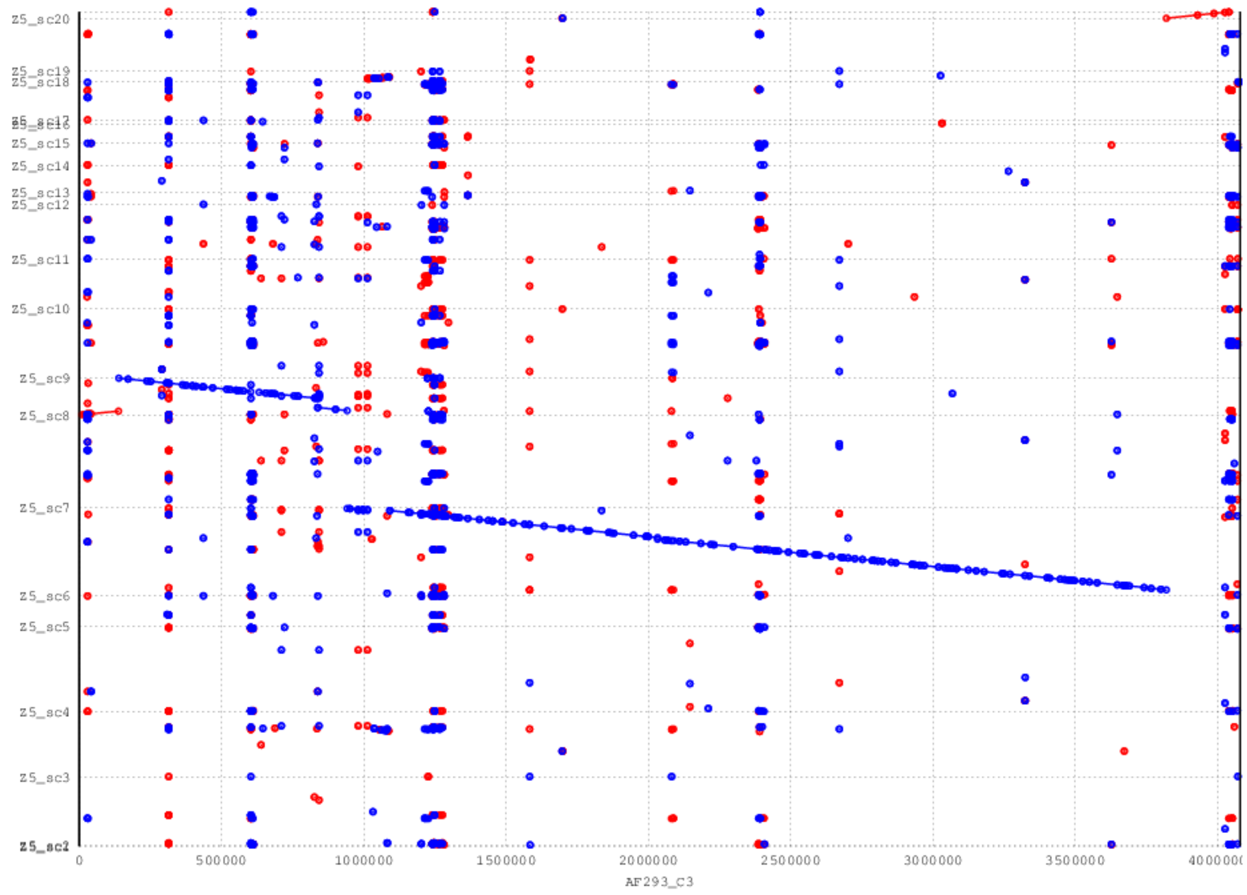

d: scaffolds matched to chromosome 4 of af293 (y-axis: all scaffolds, x-axis: chromosome 4)

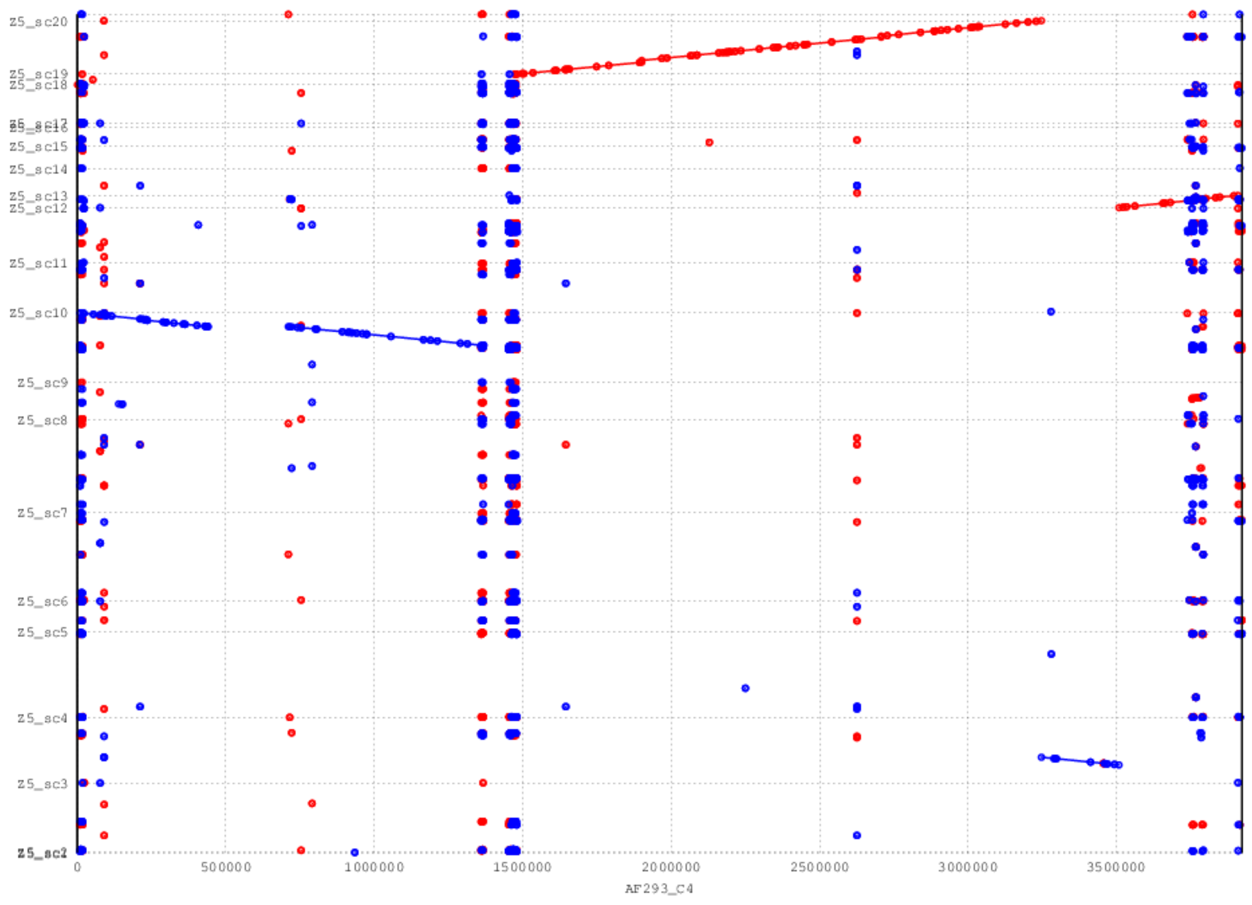

e: scaffolds matched to chromosome 5 of af293 (y-axis: all scaffolds, x-axis: chromosome5)

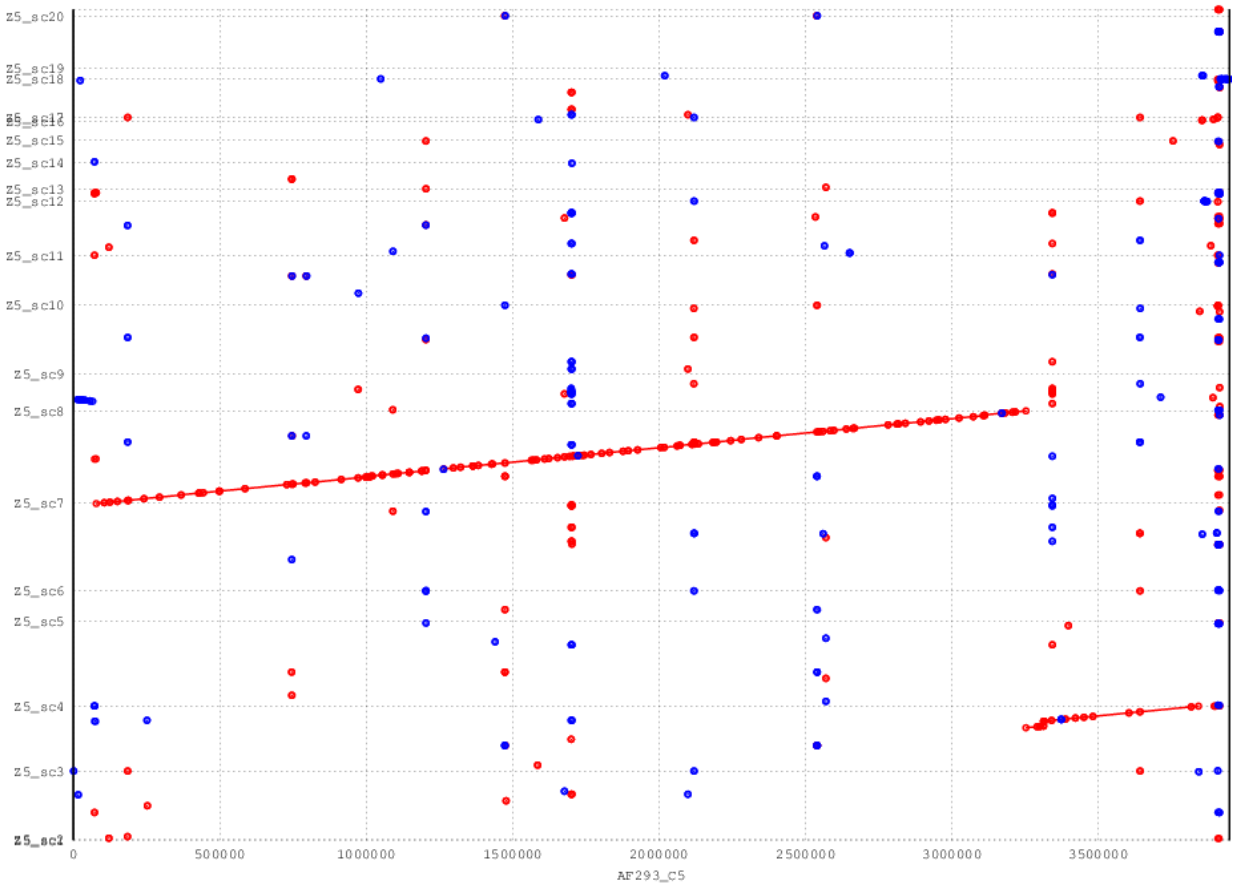

f: scaffolds matched to chromosome 6 of af293 (y-axis: all scaffolds, x-axis: chromosome6)

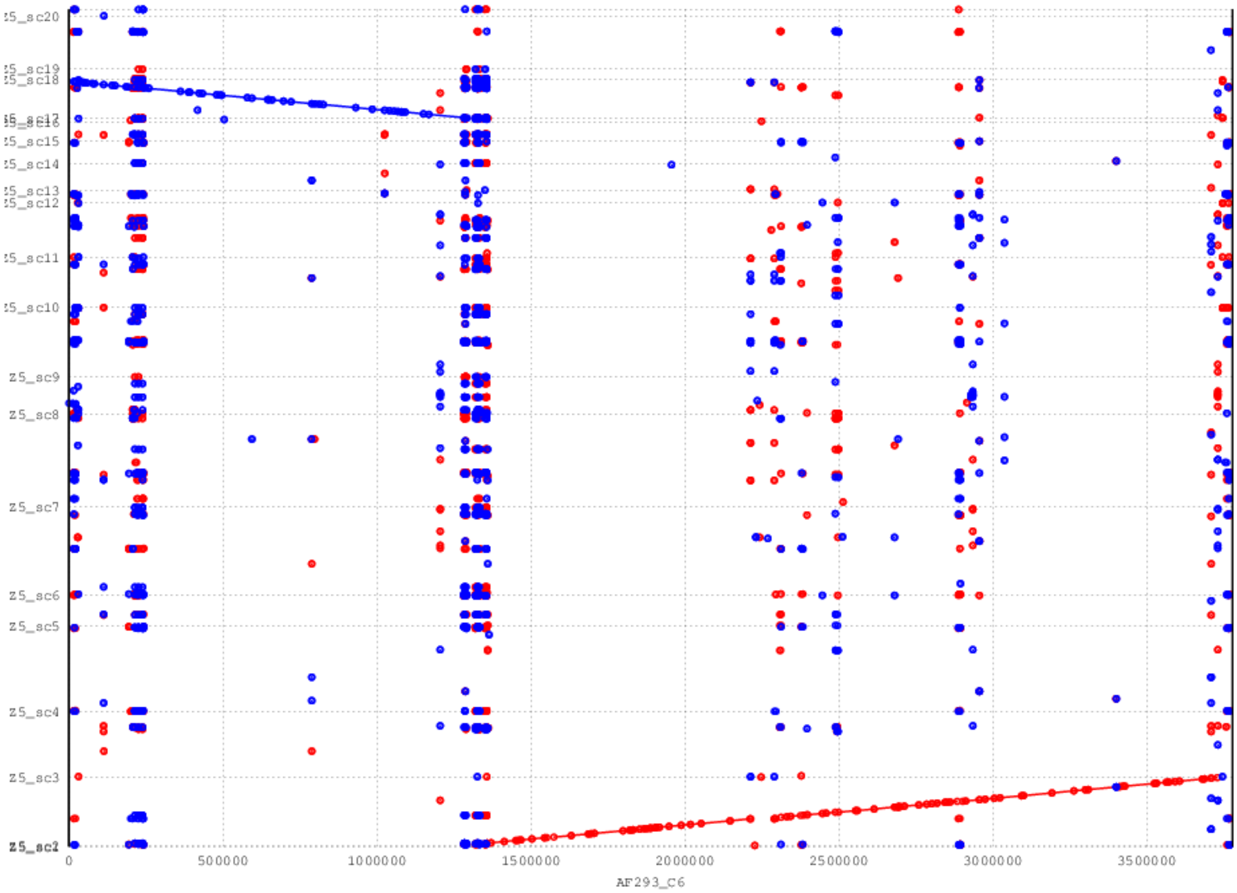

g: **scaffolds matched to chromosome 7 of af293** (y-axis: all scaffolds, x-axis: chromosome 7)

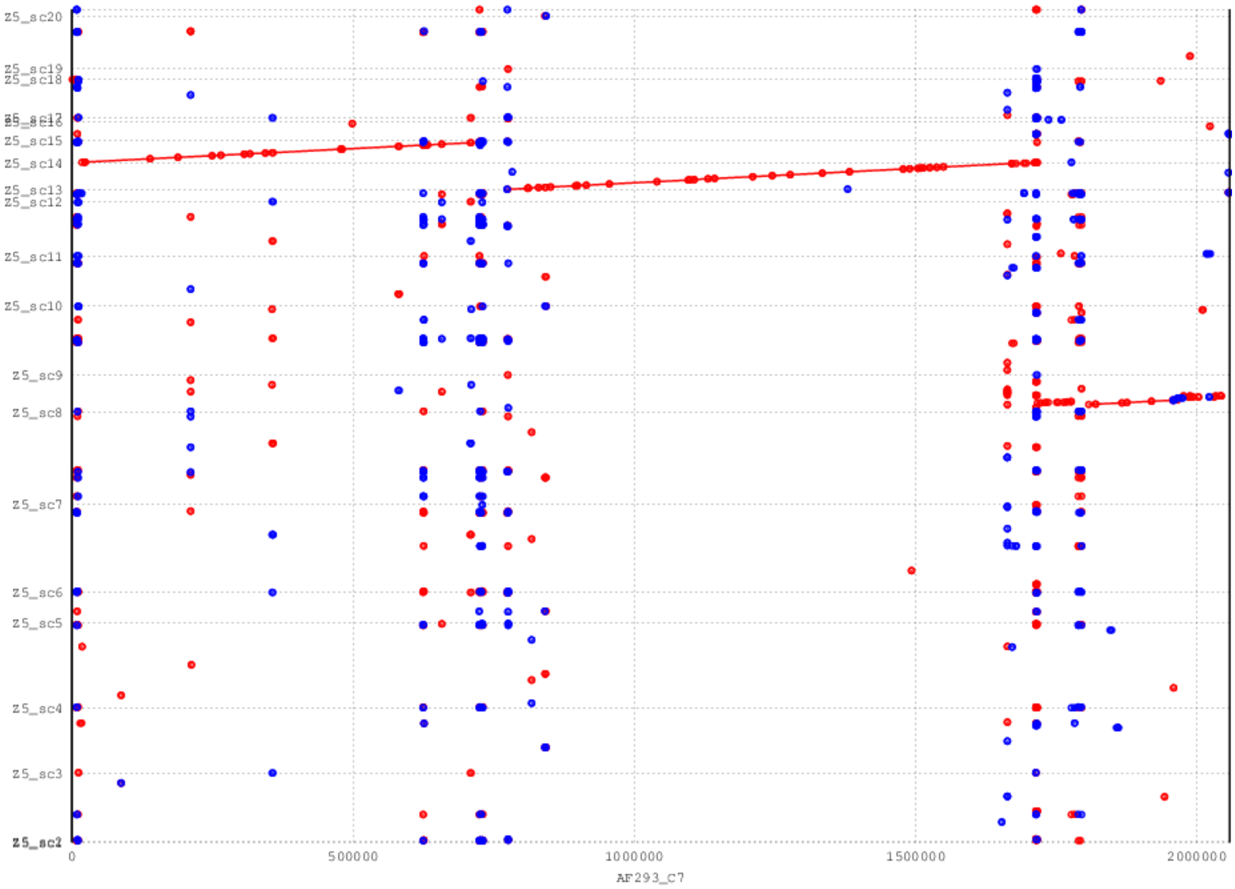

h: **scaffolds matched to chromosome 8 of af293** (y-axis: all scaffolds, x-axis: chromosome 8)

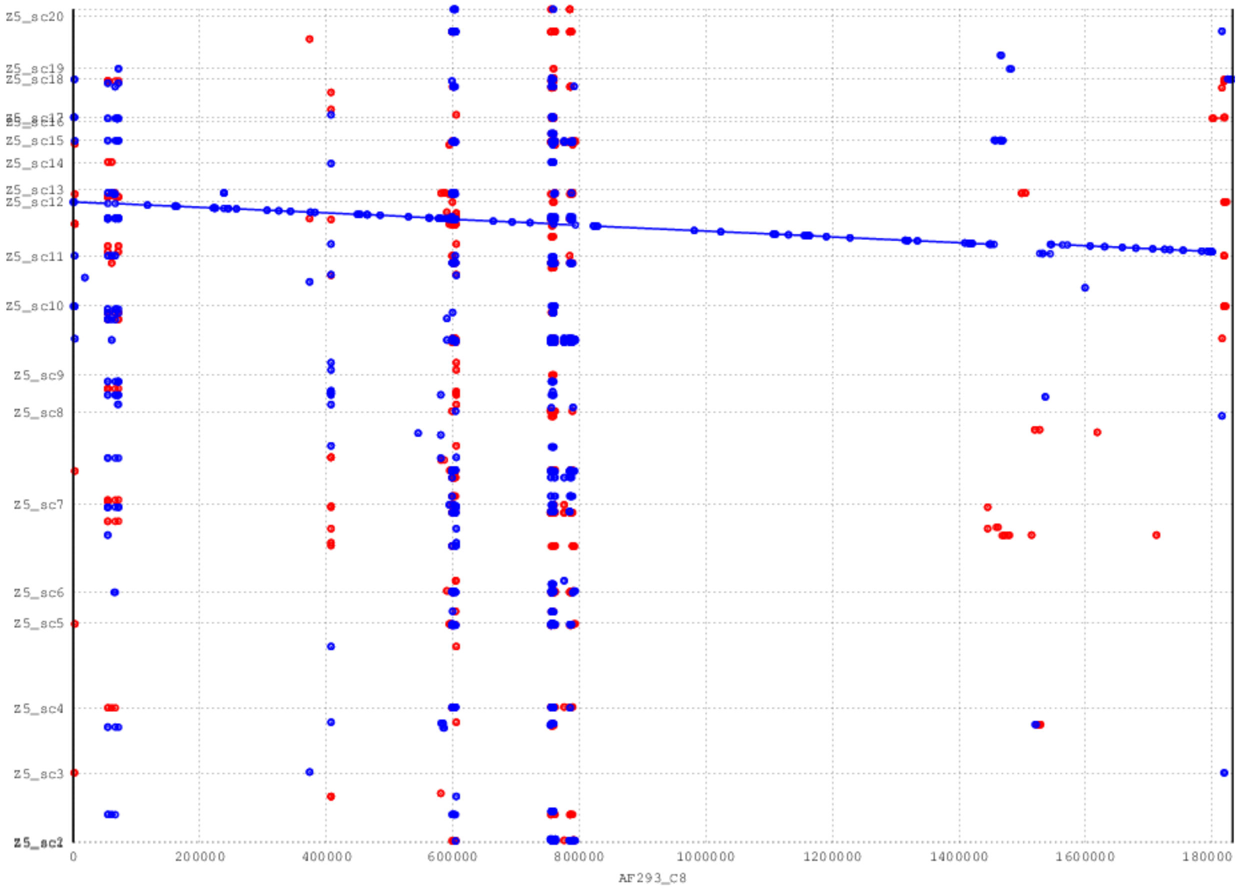

Supplement: Additional file 2: — Scaffolds matching. All Z5 scaffolds were matched into the chromosomes of A. fumigatus AF293. [file 12864_2015_1658_MOESM2_ESM.pdf]
