# Supplementary material for: Genome-wide transcriptomic analysis of a superior biomass-degrading strain of A. fumigatus revealed active lignocellulose-degrading genes
Source: BMC Genomics. 2015 Jun 16;16(1):459. doi: 10.1186/s12864-015-1658-2 (PMC4469458; doi:10.1186/s12864-015-1658-2)
Supplement: Additional file 9: — Maps of scaffolds. The map of scaffold 2 was shown by Fig. 4 with the detailed descriptions, and this file contained the maps for the remained scaffolds. [file 12864_2015_1658_MOESM9_ESM.docx]

**Additional file 9: Maps of scaffolds**

**Scaffold 3:**

**
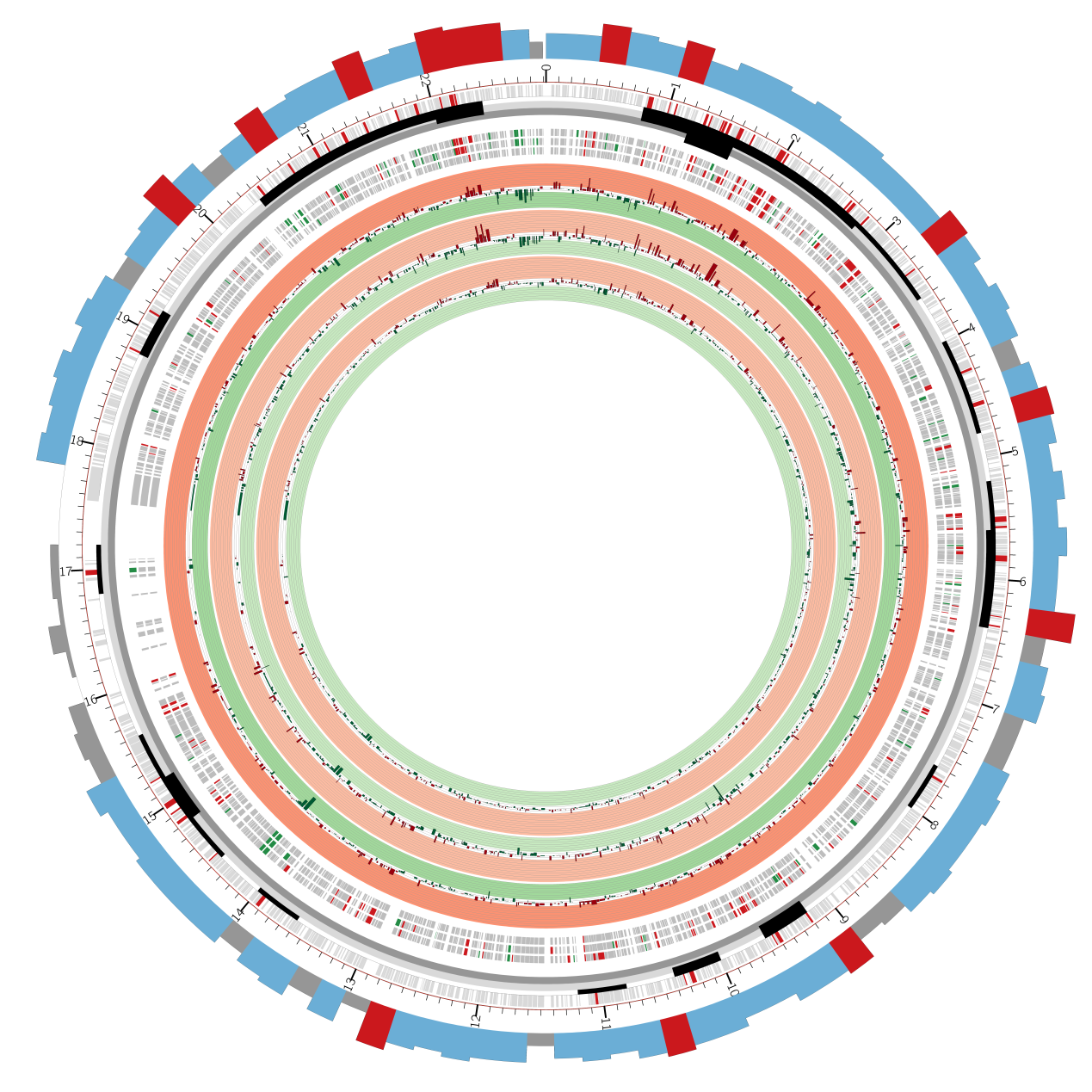
**

**Scaffold 4**

**
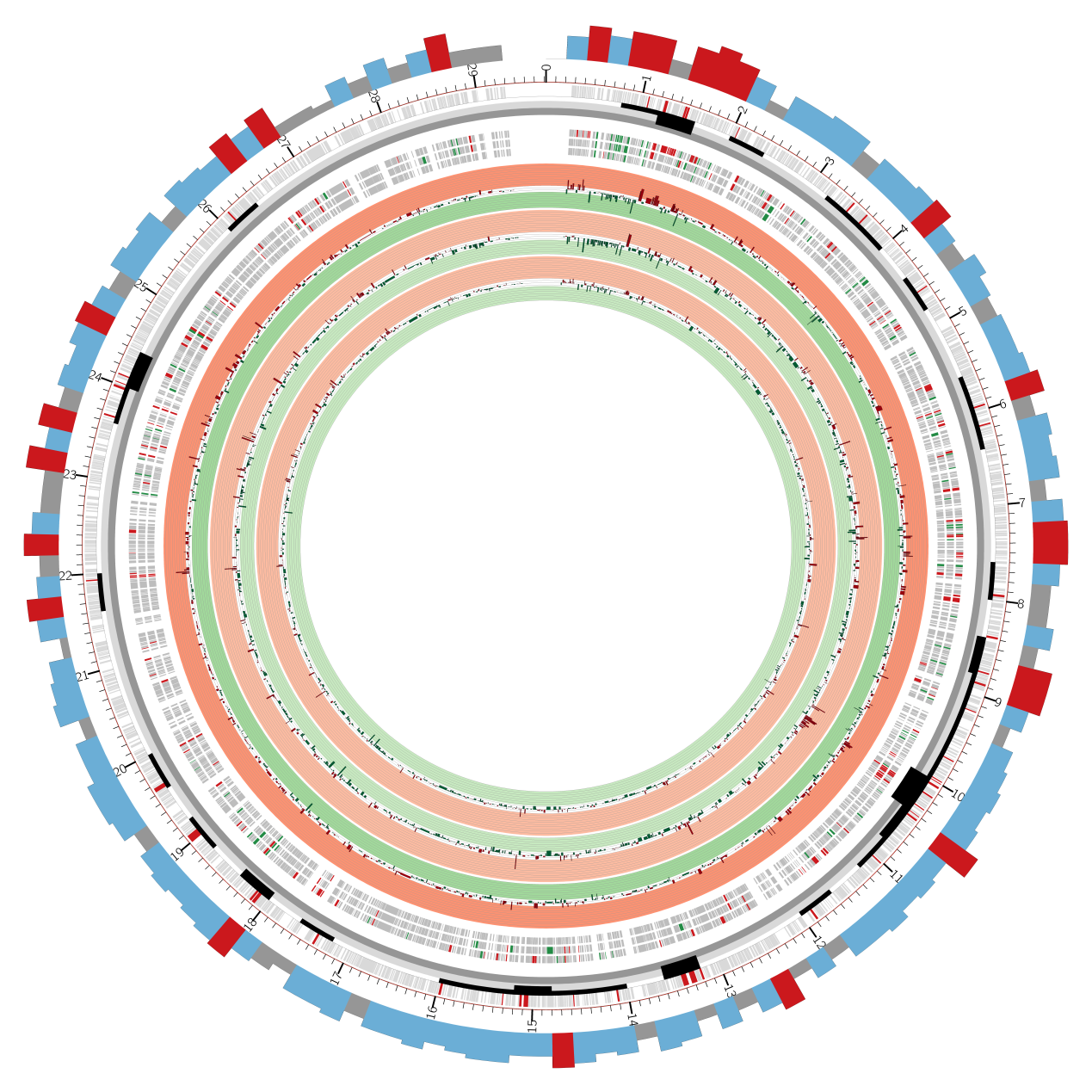
**

**Scaffold 5**

**
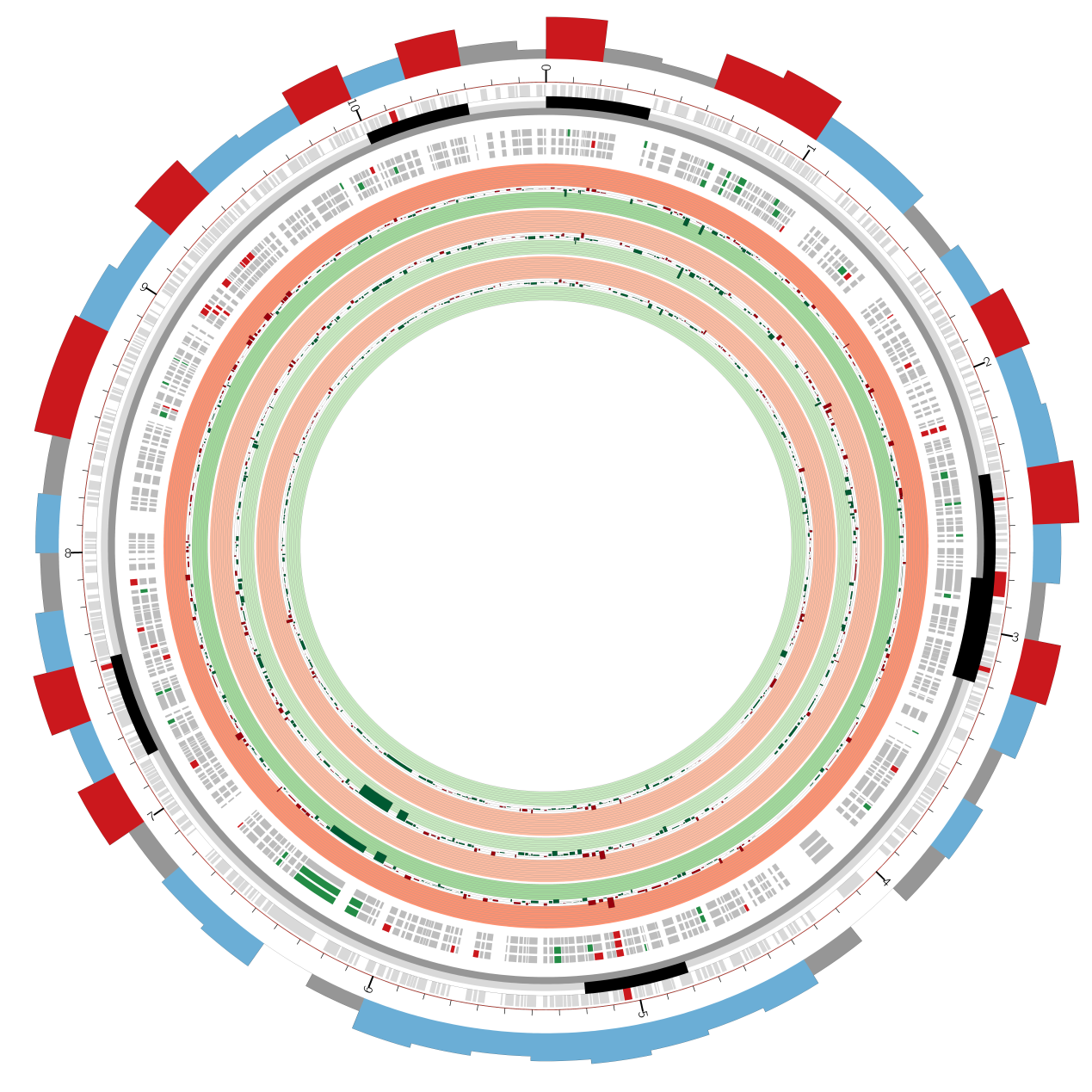
**

**Scaffold 6:**

**
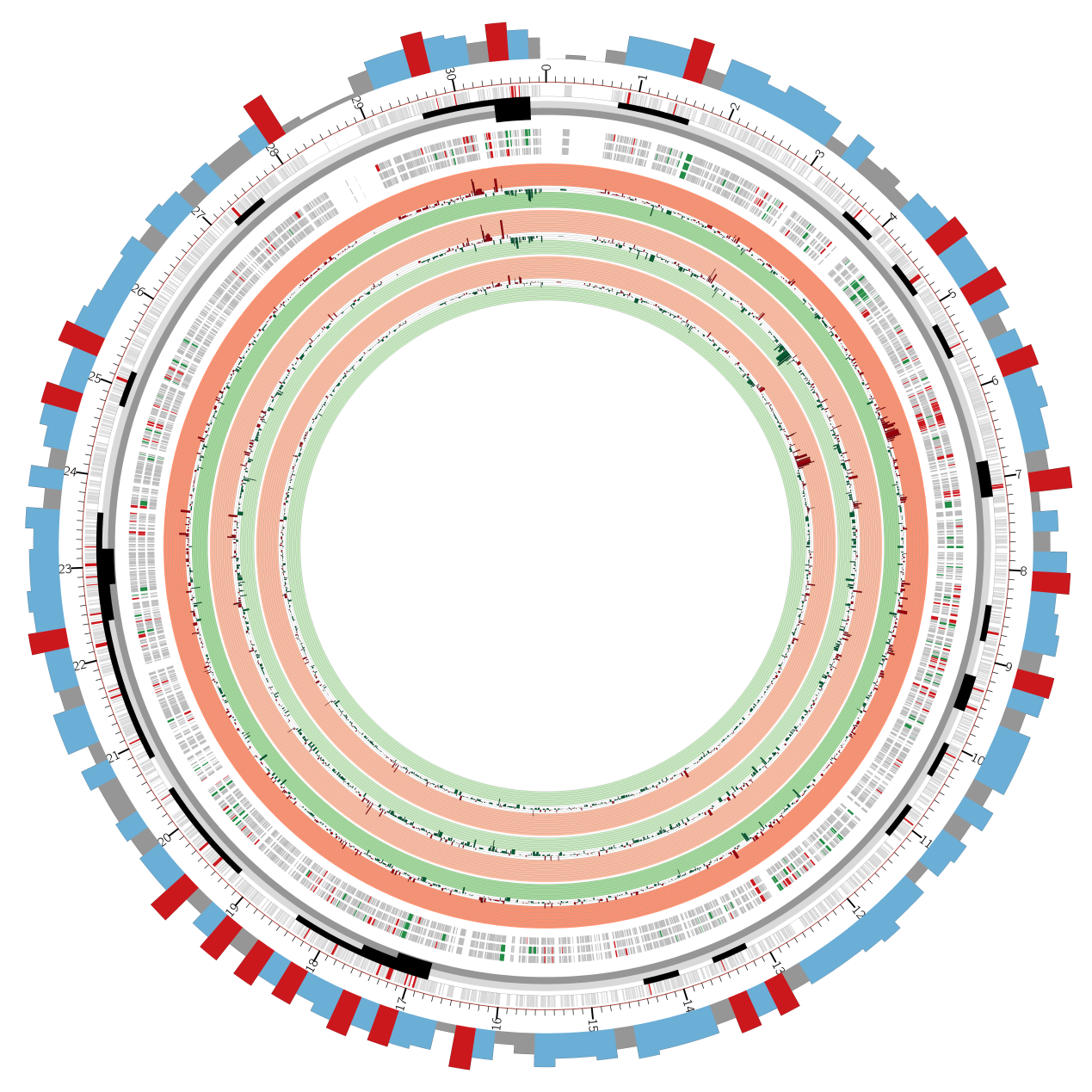
**

**Scaffold 7:**

**
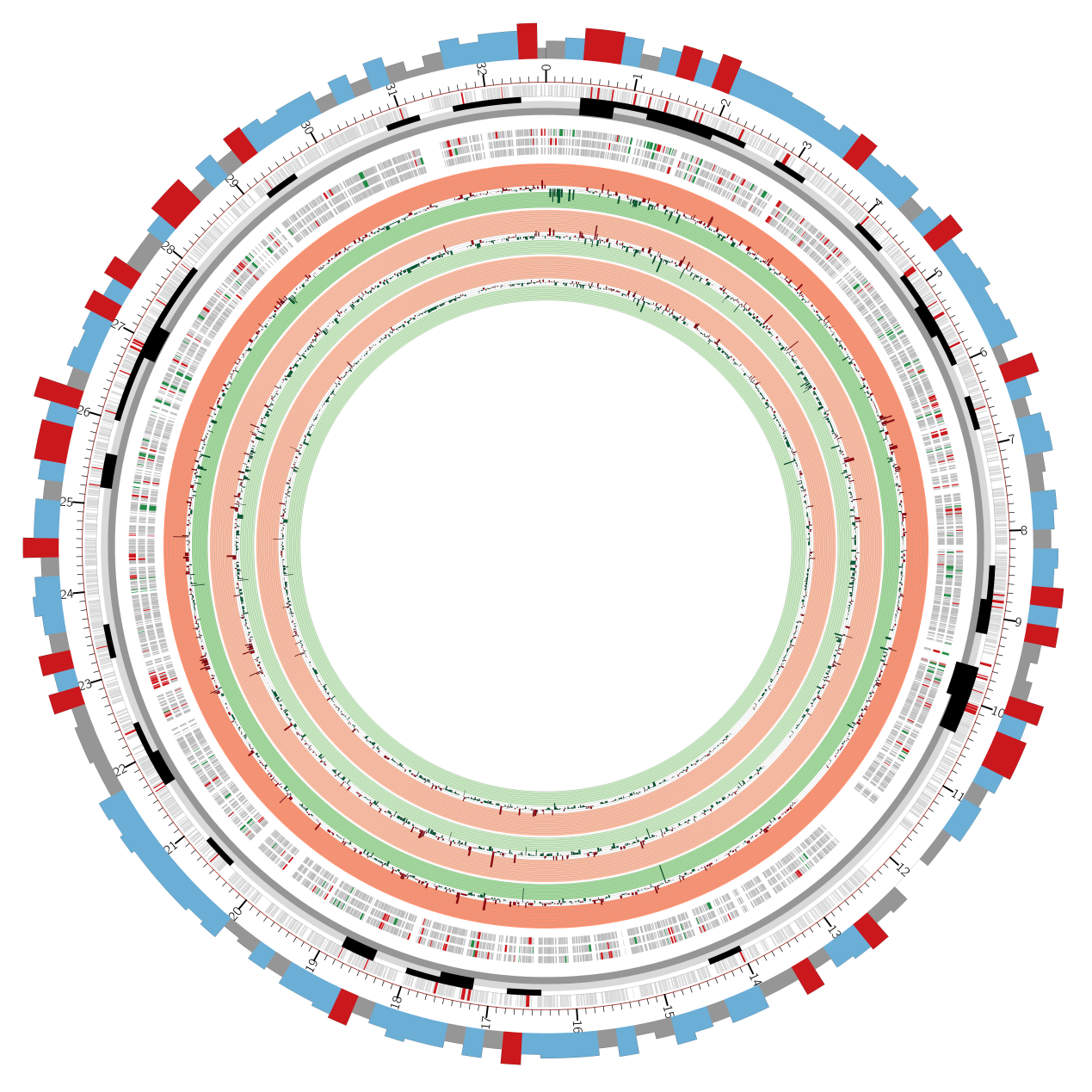
**

**Scaffold 8:**

**
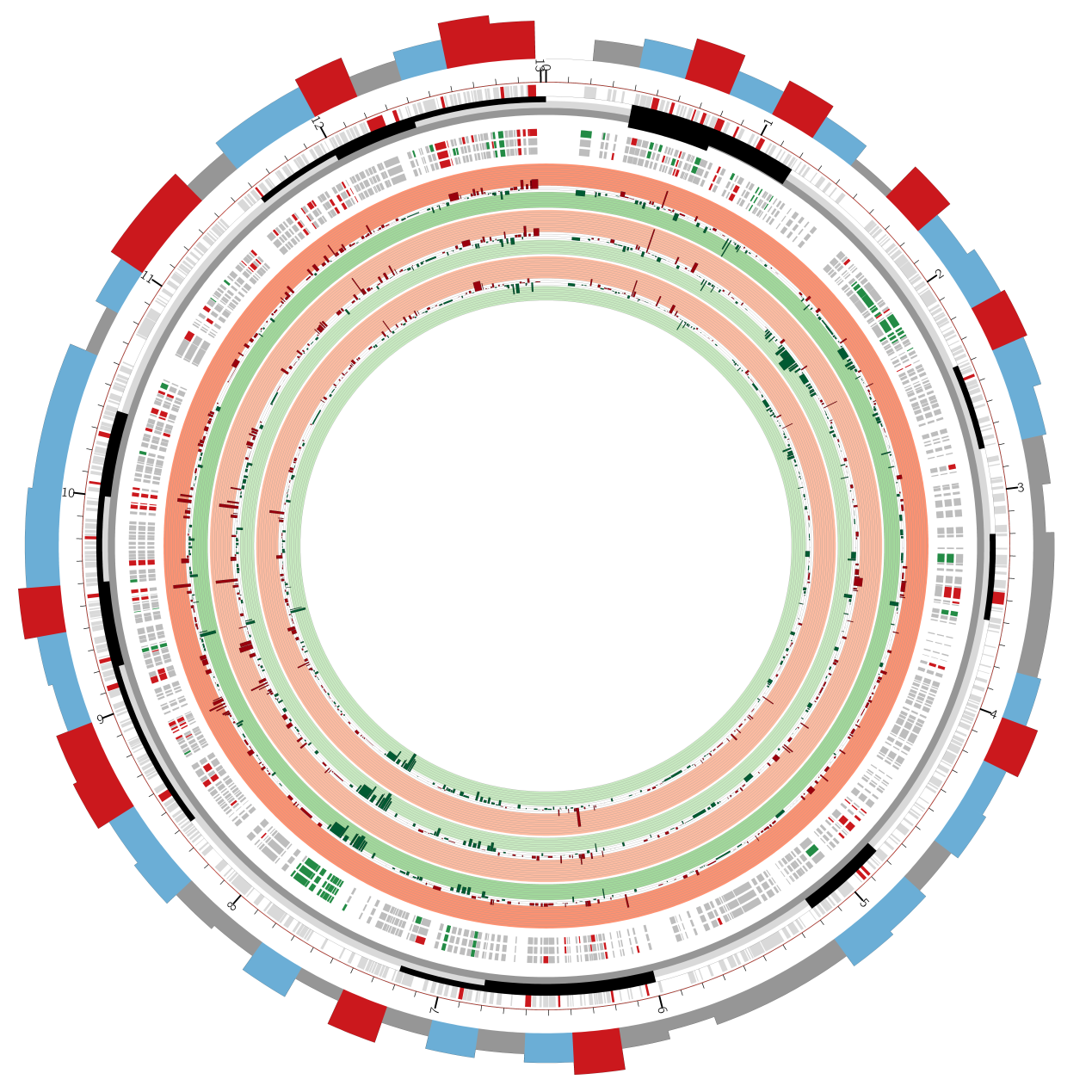
**

**Scaffold 9:**

**
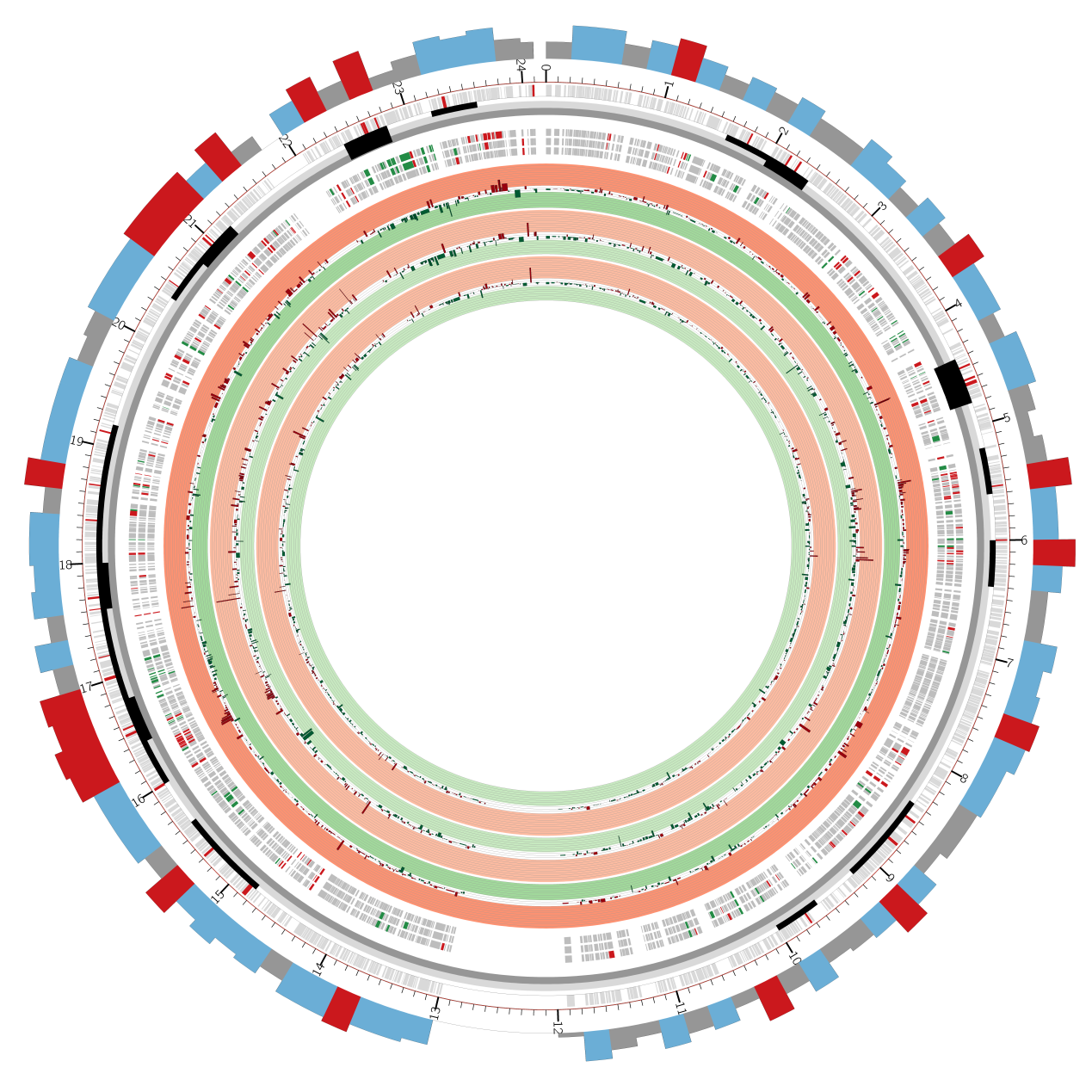
**

**Scaffold 10:**

**
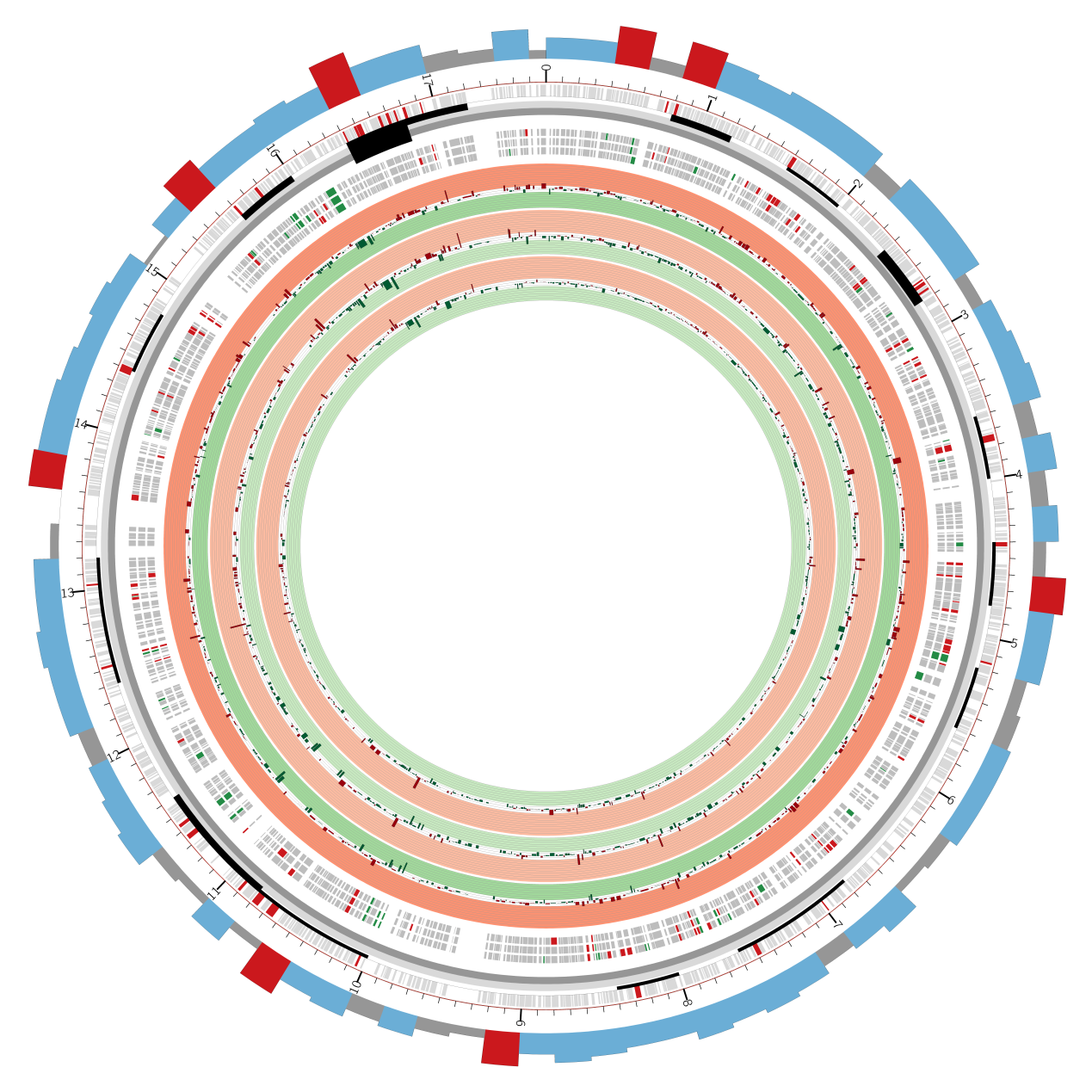
**

**Scaffold 11:**

**
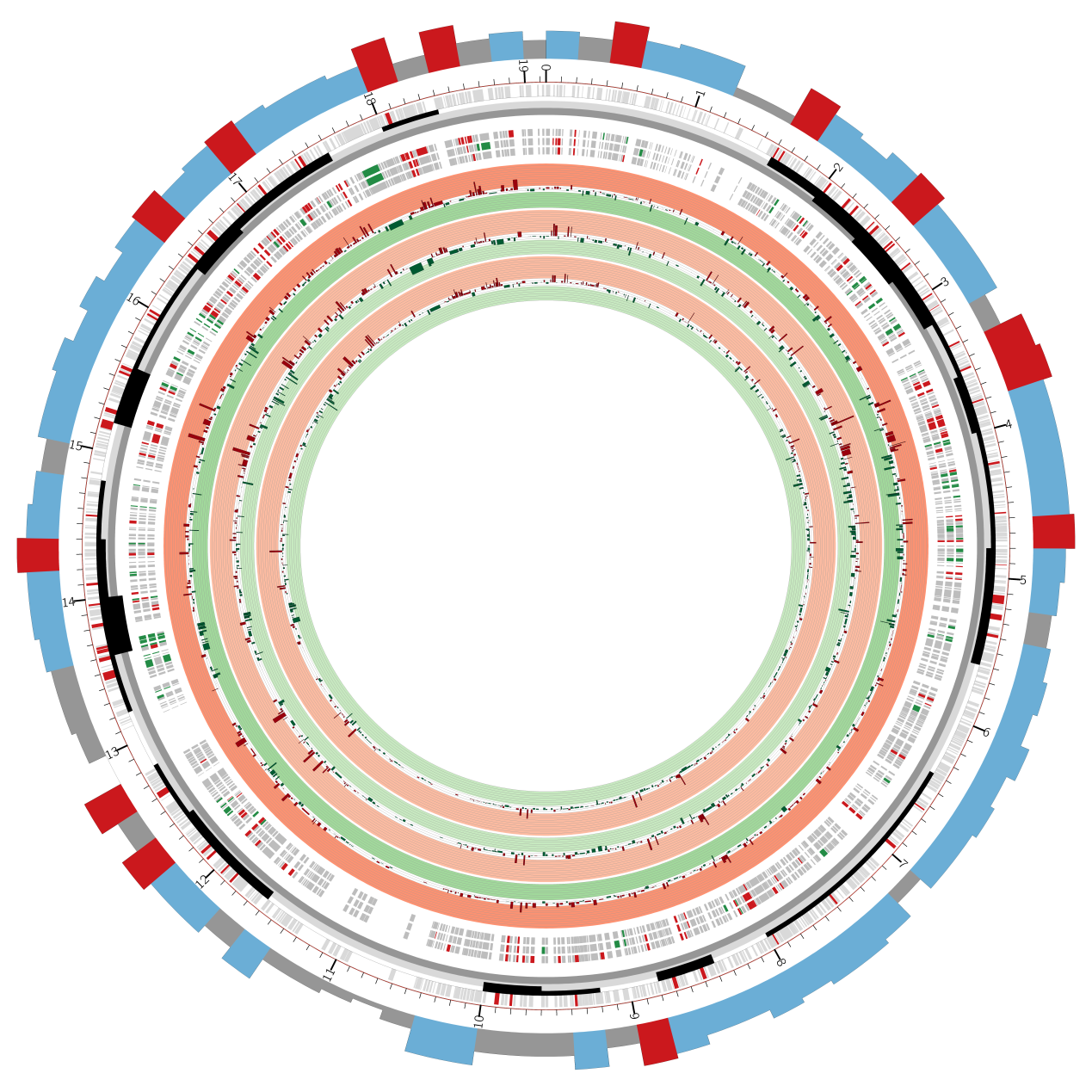
**

**Scaffold 12:**

**
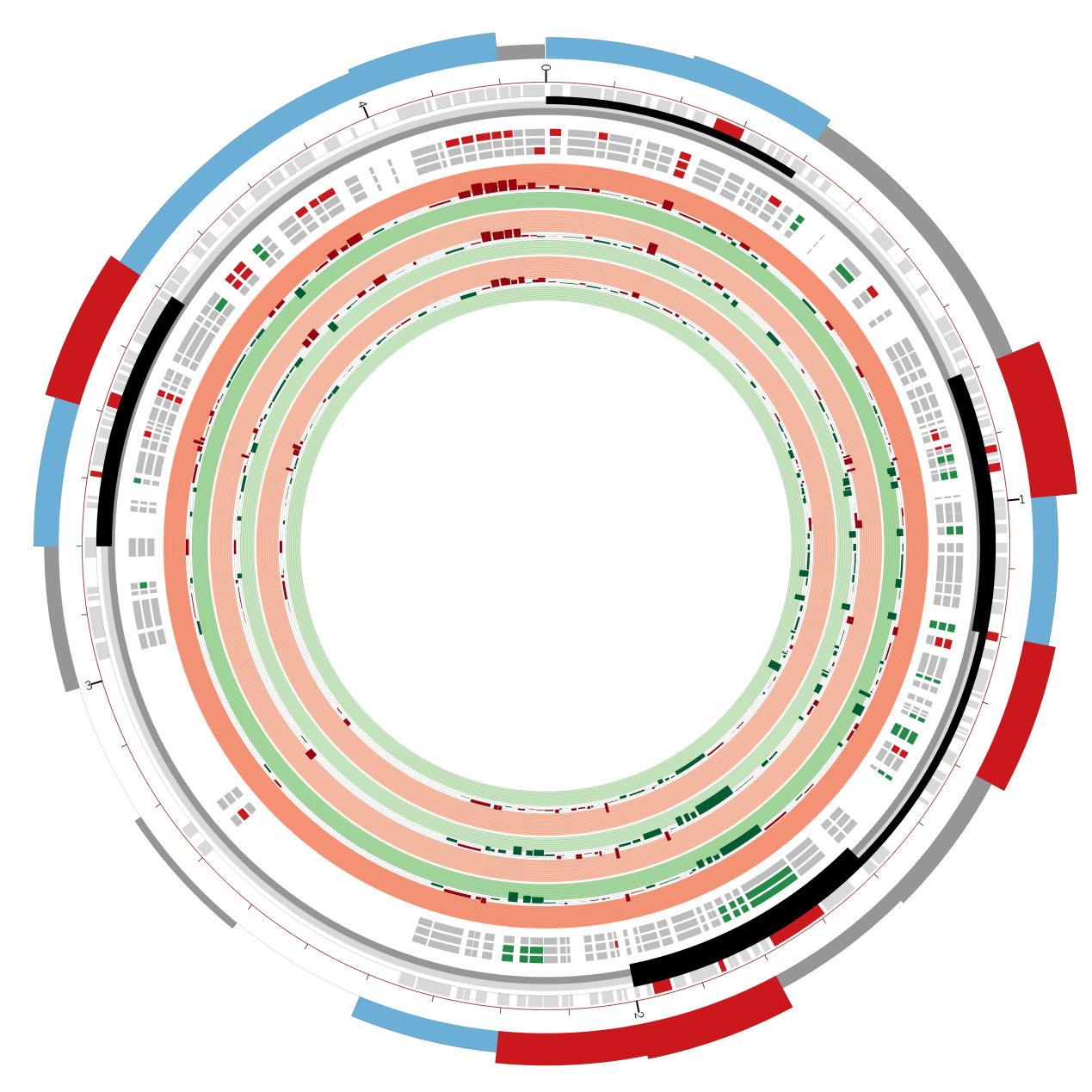
**

**Scaffold 13:**

**
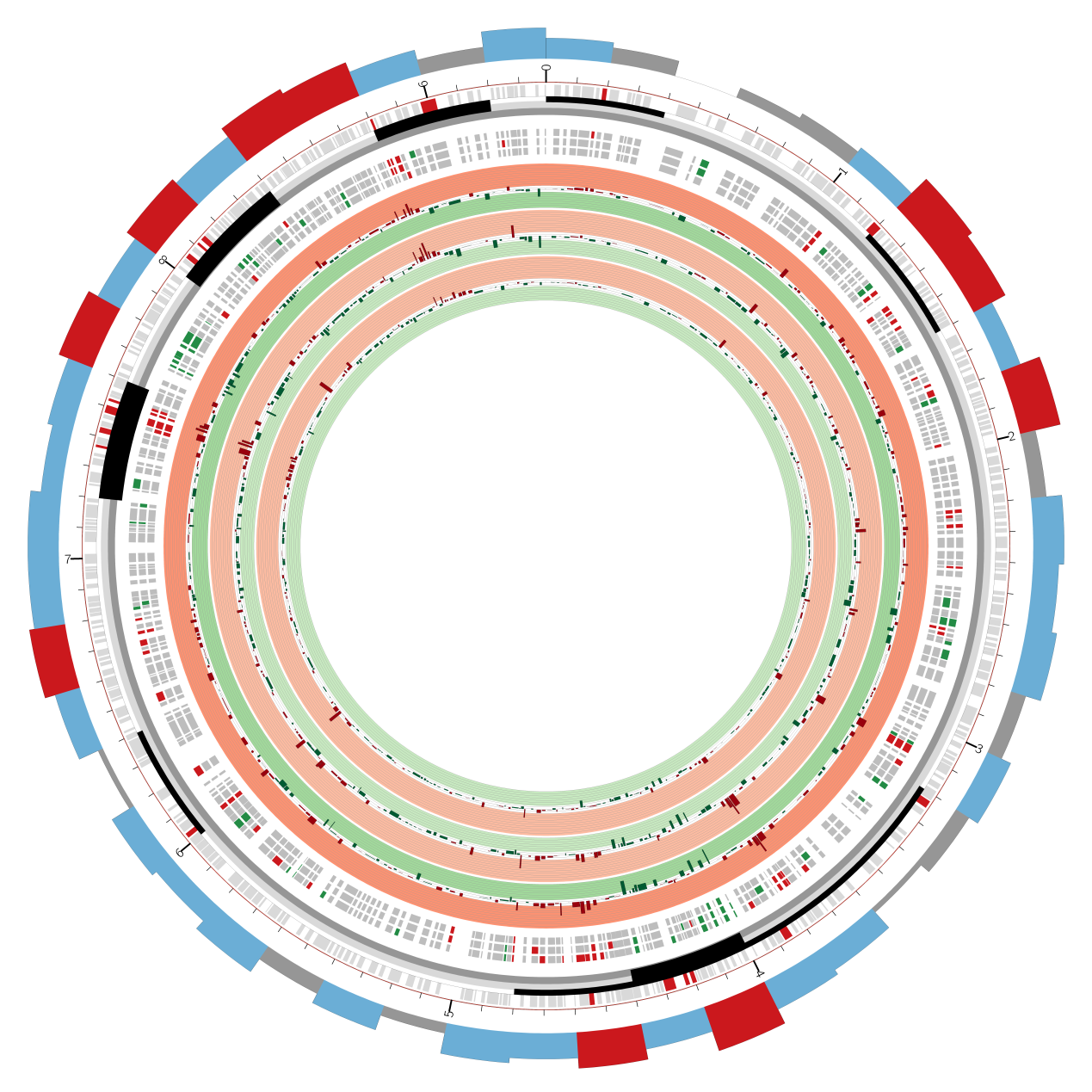
**

**Scaffold 14:**

**
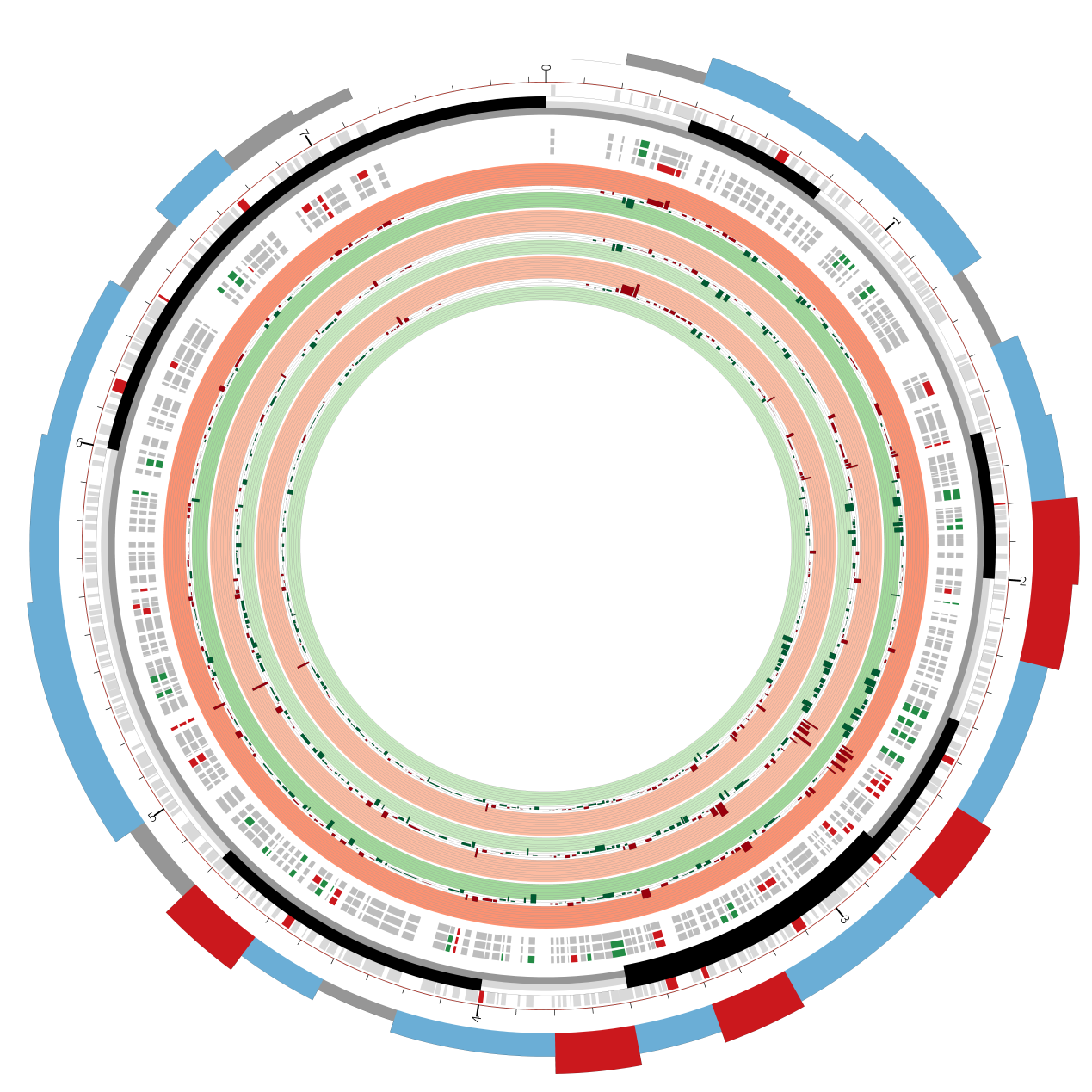
**

**Scaffold 15:**

**
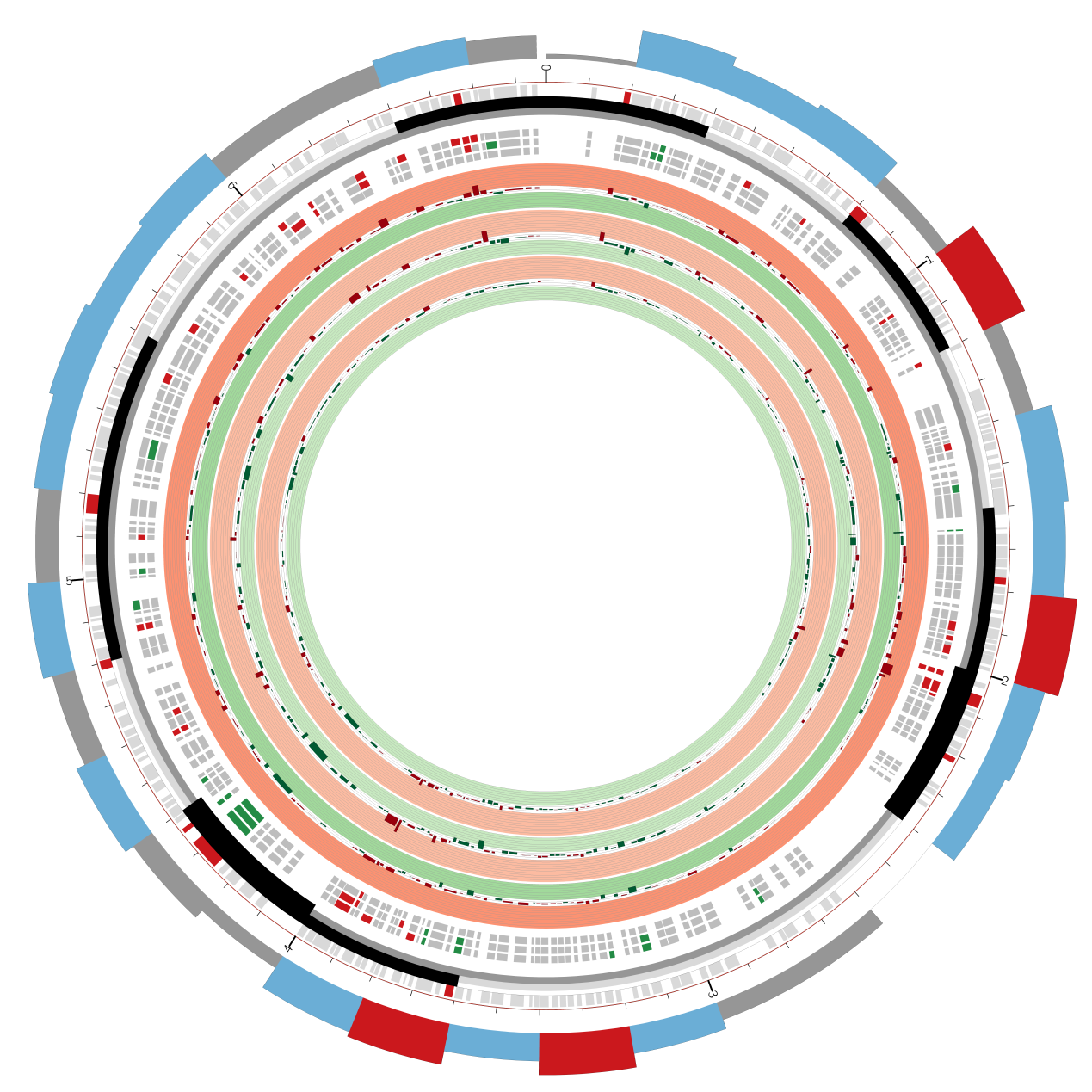
**

**Scaffold 16:**

**
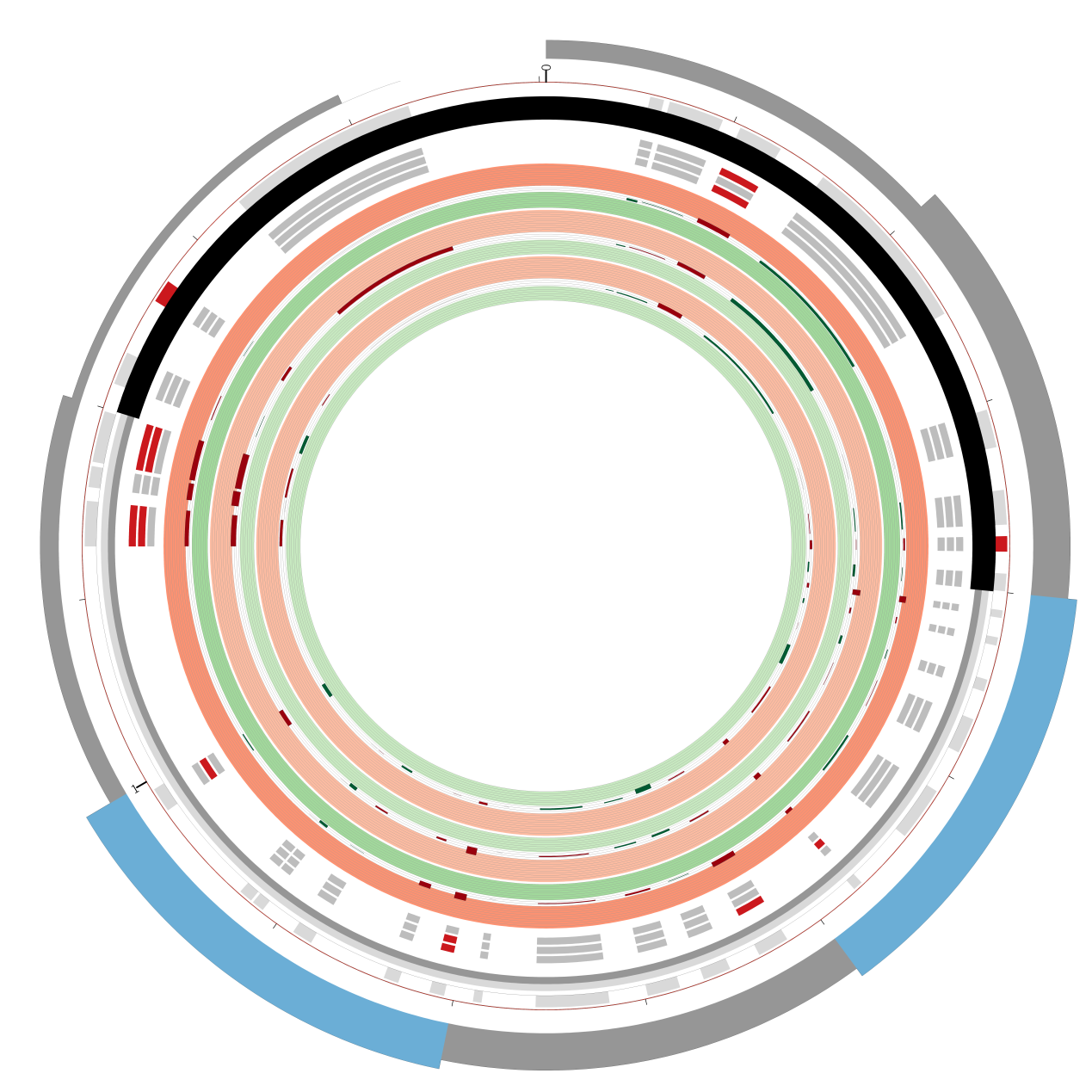
**

**Scaffold 17:**

**
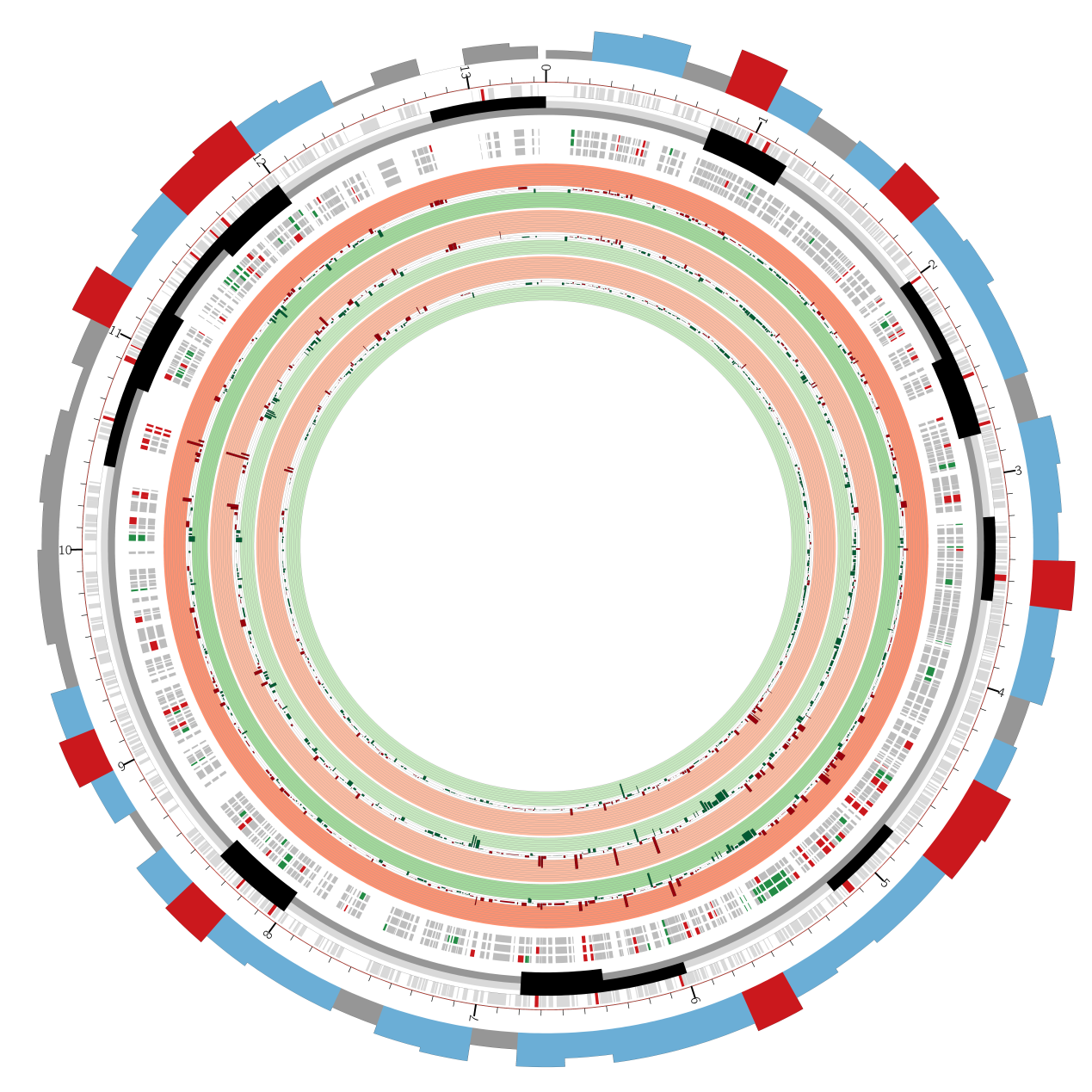
**

**Scaffold 18:**

**
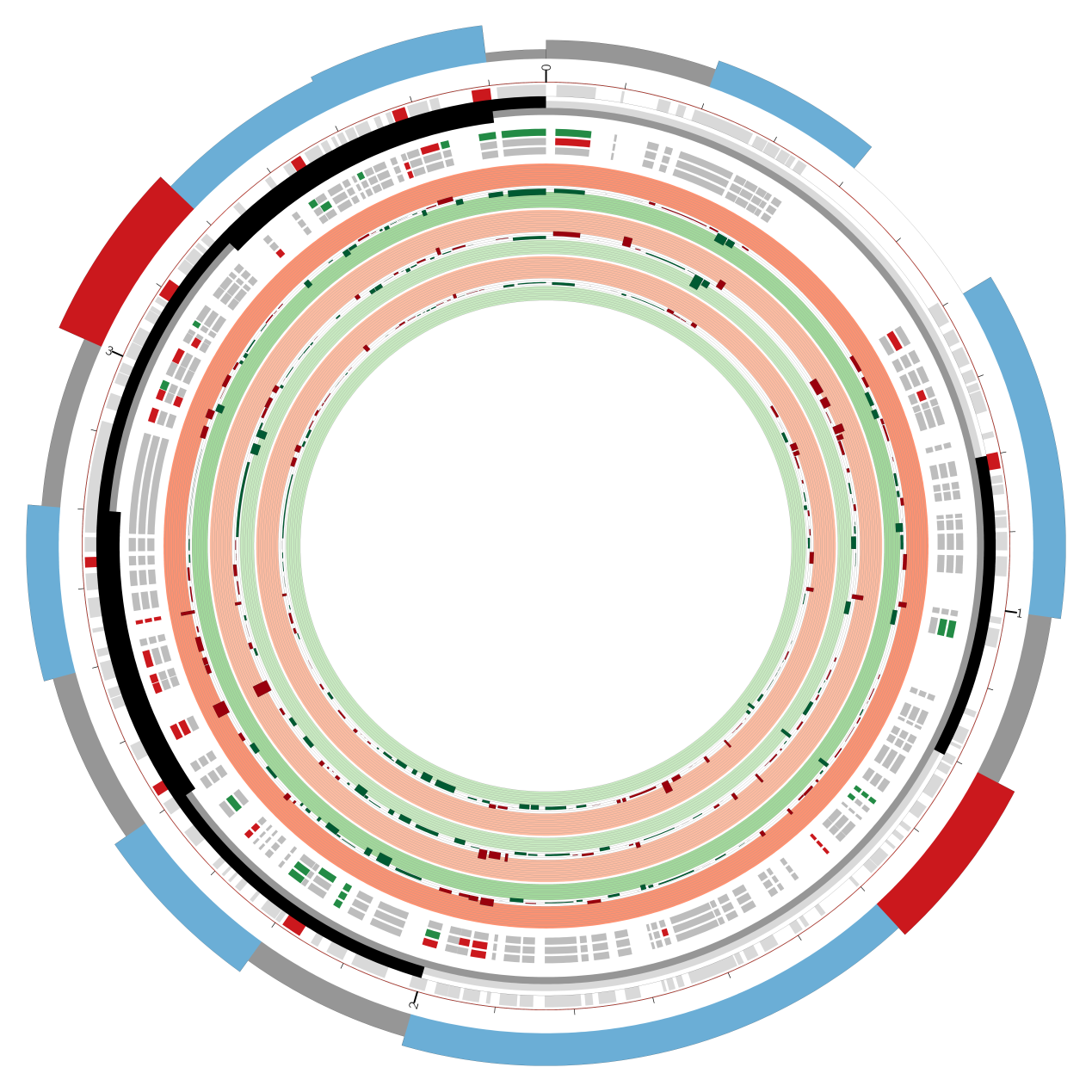
**

**Scaffold 19:**

**
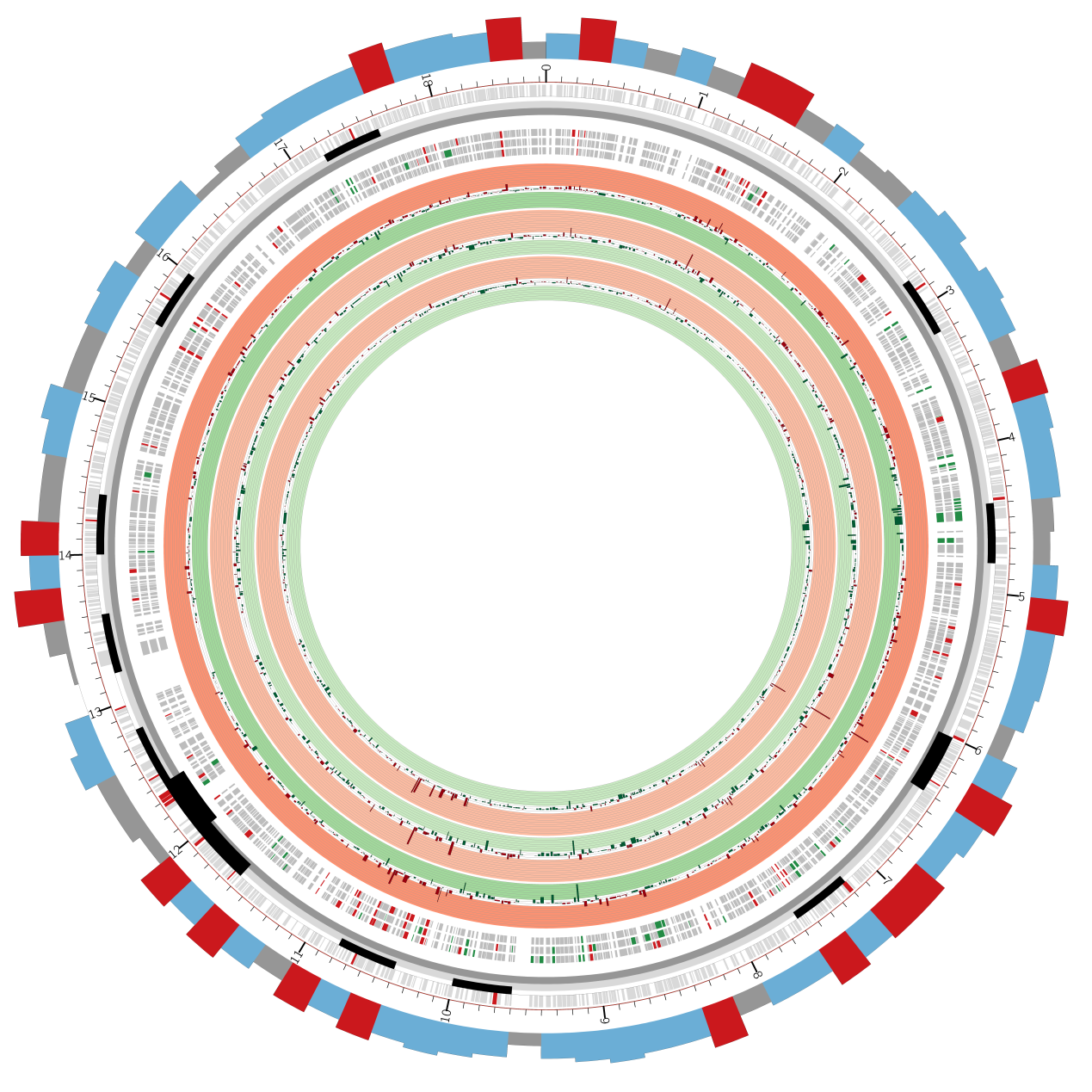
**

**Scaffold 20:**

**
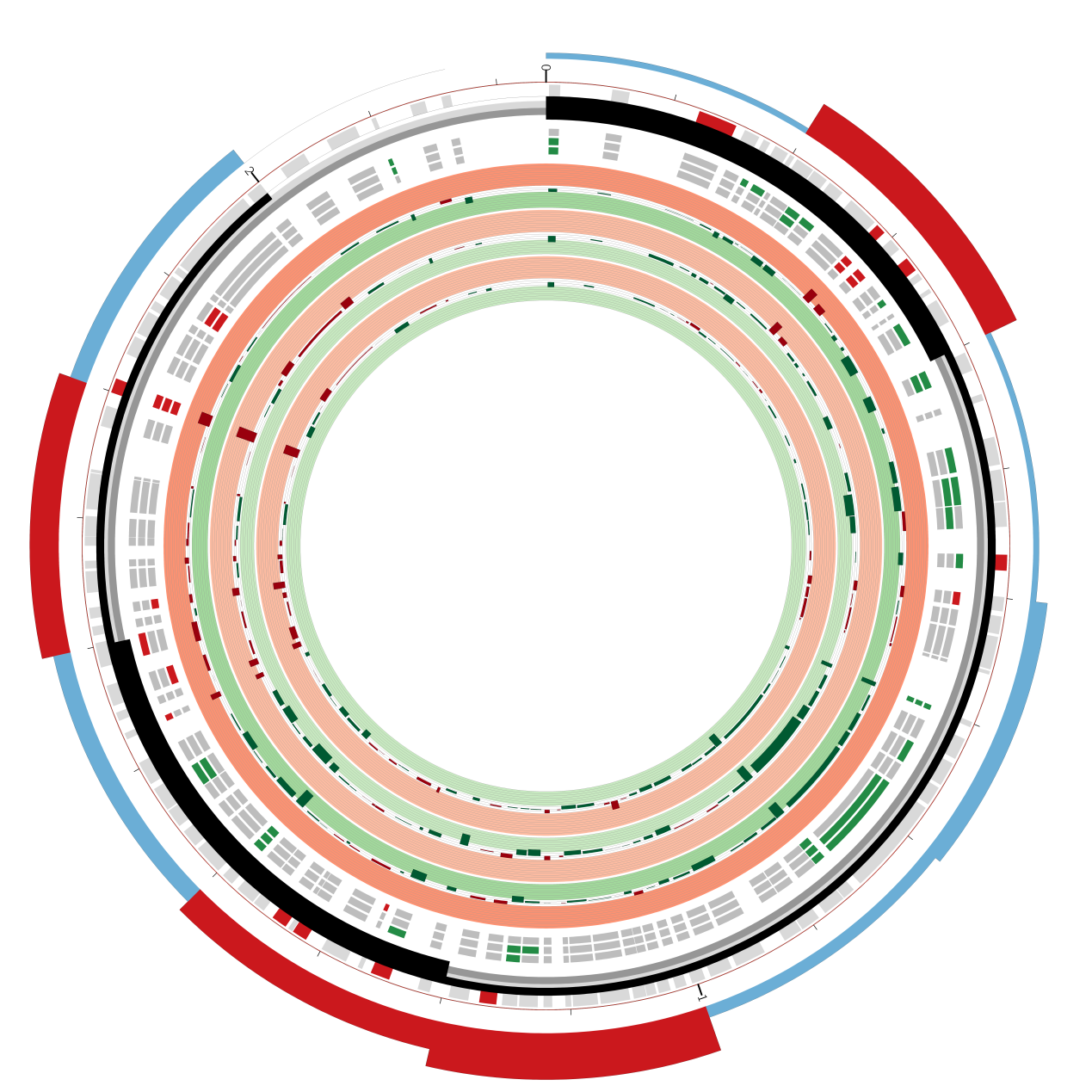
**
