# Supplementary material for: Genome-wide transcriptomic analysis of a superior biomass-degrading strain of A. fumigatus revealed active lignocellulose-degrading genes
Source: BMC Genomics. 2015 Jun 16;16(1):459. doi: 10.1186/s12864-015-1658-2 (PMC4469458; doi:10.1186/s12864-015-1658-2)
Supplement: Additional file 12: — GO enrichment analysis. 444, 1386 and 1711 significantly differently expressed genes (xylan to sucrose, rice straw to sucrose and cellulose to sucrose, respectively) were used to perform GO enrichment analysis. The results were shown in this file. [file 12864_2015_1658_MOESM12_ESM.pdf]

Additional file 12: GO enrichment analysis for three treatments compared to sucrose.

GO enrichment analysis of XGEP versus SGEP

GO.ID	Terms	Ann.	Sig.	Exp.	Classic
**MF**					
GO:0016798	hydrolase activity, acting on glycosyl bonds	278	35	13.28	8.6e-08
GO:0004553	hydrolase activity, hydrolyzing O-glycosyl compounds	241	32	11.51	9.3e-08
GO:0022857	transmembrane transporter activity	375	42	17.91	1.2e-07
GO:0005215	transporter activity	444	46	21.21	2.9e-07
GO:0031176	endo-1,4-beta-xylanase activity	7	5	0.33	4.7e-06
GO:0022891	substrate-specific transmembrane transporter activity	302	33	14.42	5.3e-06
GO:0022892	Substrate-specific transporter activity	326	34	15.57	1.1e-05
GO:0008554	sodium-exporting ATPase activity, phosphorylative mechanism	3	3	0.14	0.00011
GO:0030246	carbohydrate binding	61	11	2.91	0.00012
GO:0050364	tryptophan dimethylallyltransferase activity	7	4	0.33	0.00016
GO:0030248	cellulose binding	29	7	1.39	0.00033
GO:0046857	oxidoreductase activity, acting on other nitrogenous compounds as donors, with NAD or NADP as acceptor	4	3	0.19	0.00042
GO:0000036	ACP phosphopantetheine attachment site binding involved in fatty acid biosynthetic process	41	8	1.96	0.00059
GO:0044620	ACP phosphopantetheine attachment site binding	41	8	1.96	0.00059
GO:0051192	prosthetic group binding	41	8	1.96	0.00059
GO:0019842	vitamin binding	42	8	2.01	0.00070
GO:0001871	pattern binding	34	7	1.62	0.00093
GO:0030247	polysaccharide binding	34	7	1.62	0.00093
**BP**					
GO:0005975	Carbohydrate metabolic process	397	50	19.07	8.9e-11
GO:0008643	Carbohydrate transport	68	18	3.42	3.2e-09
GO:0044765	Single-organism transport	721	64	36.28	1.8e-06
GO:0016052	Carbohydrate catabolic process	152	22	7.65	5.6e-06
GO:0055085	Transmembrane transport	588	53	29.59	1.1e-05
GO:0000272	Polysaccharide catabolic process	95	15	4.78	6.6e-05
GO:0042732	D-xylose metabolic process	3	3	0.15	0.00013
GO:0044262	Cellular carbohydrate metabolic process	69	12	3.47	0.00014
GO:0009820	Alkaloid metabolic process	7	4	0.35	0.00019
GO:0005976	Polysaccharide metabolic process	107	15	5.38	0.00026
GO:0044723	Single-organism carbohydrate metabolic process	137	17	6.89	0.00045
GO:0045491	Xylan metabolic process	23	6	1.16	0.00075
GO:0045493	Xylan catabolic process	23	6	1.16	0.00075
GO:0010410	Hemicellulose metabolic process	24	6	1.21	0.00096
**CC**					
GO:0031224	Intrinsic to membrane	943	73	41.38	8.1e-09
GO:0005576	Extracellular region	136	22	5.79	4.9e-08
GO:0016021	Integral to membrane	917	69	40.23	1.2e-07
GO:0044425	Membrane part	1026	73	45.02	4.1e-07
GO:0016020	membrane	1269	81	55.68	7.1e-06

Ann. means all the annotated genes in that group; **Sig.** means significant genes which were differently expressed in xylan compared to sucrose; **Exp.** Means expectation values when that group was enriched. GO enrichment analysis was done in three different aspects (**MF**, molecular function; **BP**, biology process; **CC**, cellular location). **Classic**, the classic algorithm used by the topGO package.

GO enrichment analysis of RGEP versus SGEP

GO.ID	Terms	Ann.	Sig.	Exp.	classic
**MF**					
GO:0004553	hydrolase activity, hydrolyzing O-glycosyl compounds	241	87	36.5	1.90E-16
GO:0016798	hydrolase activity, acting on glycosyl bonds	278	92	42.1	1.30E-14
GO:0030246	carbohydrate binding	61	35	9.24	2.60E-14
GO:0030248	cellulose binding	29	22	4.39	4.00E-13
GO:0001871	pattern binding	34	23	5.15	5.80E-12
GO:0030247	polysaccharide binding	34	23	5.15	5.80E-12
GO:0003824	catalytic activity	4032	680	610.66	1.90E-08
GO:0016491	oxidoreductase activity	1059	221	160.39	1.90E-08
GO:0005506	iron ion binding	75	29	11.36	5.00E-07
GO:0048037	cofactor binding	400	94	60.58	3.30E-06
GO:0050662	coenzyme binding	260	67	39.38	3.70E-06
GO:0008810	cellulase activity	17	11	2.57	4.70E-06
GO:0031176	endo-1,4-beta-xylanase activity	7	6	1.06	7.30E-05
GO:0016614	oxidoreductase activity, acting on CH-OH...	232	57	35.14	8.60E-05
GO:0016616	oxidoreductase activity, acting on the C...	212	53	32.11	9.30E-05
GO:0004091	carboxylesterase activity	28	12	4.24	0.00039
GO:0046857	oxidoreductase activity, acting on other...	4	4	0.61	0.00052
GO:0008422	beta-glucosidase activity	29	12	4.39	0.00058
GO:0043169	cation binding	1291	233	195.53	0.00069
GO:0010181	FMN binding	42	15	6.36	0.0008
**BP**					
GO:0005975	carbohydrate metabolic process	379	132	59.82	1.40E-21
GO:0005976	polysaccharide metabolic process	107	49	16.89	1.30E-13
GO:0000272	polysaccharide catabolic process	95	44	14.99	1.40E-12
GO:0016052	carbohydrate catabolic process	152	59	23.99	2.60E-12
GO:0008643	carbohydrate transport	68	30	10.73	2.30E-08
GO:0055114	oxidation-reduction process	1002	216	158.15	4.20E-08
GO:0055085	transmembrane transport	588	134	92.81	1.40E-06
GO:0010383	cell wall polysaccharide metabolic process	25	14	3.95	4.30E-06
GO:0044723	single-organism carbohydrate metabolic process	137	42	21.62	7.40E-06
GO:0044262	cellular carbohydrate metabolic process	69	26	10.89	7.40E-06
GO:0045491	xylan metabolic process	23	13	3.63	8.40E-06
GO:0045493	xylan catabolic process	23	13	3.63	8.40E-06
GO:0009057	macromolecule catabolic process	189	53	29.83	9.20E-06
GO:0010410	hemicellulose metabolic process	24	13	3.79	1.60E-05
GO:0071554	cell wall organization or biogenesis	50	20	7.89	3.10E-05
GO:0044042	glucan metabolic process	29	14	4.58	4.00E-05
GO:0044036	cell wall macromolecule metabolic process	44	18	6.94	5.40E-05
GO:0009251	glucan catabolic process	23	12	3.63	5.50E-05
GO:0010393	galacturonan metabolic process	23	12	3.63	5.50E-05
GO:0045488	pectin metabolic process	23	12	3.63	5.50E-05
GO:0045490	pectin catabolic process	23	12	3.63	5.50E-05
GO:0030243	cellulose metabolic process	20	11	3.16	6.00E-05
GO:0030245	cellulose catabolic process	20	11	3.16	6.00E-05
GO:0051275	beta-glucan catabolic process	20	11	3.16	6.00E-05
GO:0006073	cellular glucan metabolic process	27	13	4.26	7.90E-05
GO:0044264	cellular polysaccharide metabolic process	38	16	6	9.20E-05
GO:0044765	single-organism transport	721	149	113.8	0.0001
GO:0051273	beta-glucan metabolic process	21	11	3.31	0.00011
GO:0044275	cellular carbohydrate catabolic process	35	15	5.52	0.00012
GO:0044724	single-organism carbohydrate catabolic process	72	24	11.36	0.00016
GO:0044247	cellular polysaccharide catabolic process	29	13	4.58	0.0002
GO:0044710	single-organism metabolic process	1658	303	261.69	0.00049
GO:0051704	multi-organism process	32	13	5.05	0.00064
**CC**					
GO:0005576	extracellular region	136	63	20.05	1.60E-19
GO:0031224	intrinsic to membrane	943	189	139.01	5.60E-08
GO:0016021	integral to membrane	917	181	135.18	4.80E-07
GO:0044425	membrane part	1026	190	151.24	2.70E-05
GO:0016020	membrane	1269	215	187.07	0.0026

GO enrichment analysis of CGEP versus SGEP

GO.ID	Terms	Ann.	Sig.	Exp.	classic
**MF**					
GO:0016491	oxidoreductase activity	1059	292	199.99	1.00E-14
GO:0004553	hydrolase activity, hydrolyzing O-glycosyl compounds	241	93	45.51	2.40E-13
GO:0016798	hydrolase activity, acting on glycosyl bonds	278	100	52.5	4.50E-12
GO:0001871	pattern binding	34	23	6.42	6.20E-10
GO:0030247	polysaccharide binding	34	23	6.42	6.20E-10
GO:0030248	cellulose binding	29	20	5.48	5.00E-09
GO:0030246	carbohydrate binding	61	30	11.52	7.30E-08
GO:0005506	iron ion binding	75	32	14.16	1.60E-06
GO:0004129	cytochrome-c oxidase activity	10	8	1.89	5.00E-05
GO:0015002	heme-copper terminal oxidase activity	10	8	1.89	5.00E-05
GO:0016675	oxidoreductase activity, acting on a hem...	10	8	1.89	5.00E-05
GO:0016676	oxidoreductase activity, acting on a hem...	10	8	1.89	5.00E-05
GO:0050660	flavin adenine dinucleotide binding	113	39	21.34	5.30E-05
GO:0008422	beta-glucosidase activity	29	15	5.48	6.80E-05
GO:0050662	coenzyme binding	260	73	49.1	0.00014
GO:0016616	oxidoreductase activity, acting on the C...	212	61	40.04	0.00024
GO:0003824	catalytic activity	4032	810	761.44	0.00025
GO:0048037	cofactor binding	400	103	75.54	0.00028
GO:0016614	oxidoreductase activity, acting on CH-OH...	232	65	43.81	0.00035
GO:0016705	oxidoreductase activity, acting on paire...	176	50	33.24	0.00118
**BP**					
GO:0022613	ribonucleoprotein complex biogenesis	84	53	16.39	1.40E-18
GO:0042254	ribosome biogenesis	84	53	16.39	1.40E-18
GO:0006364	rRNA processing	61	38	11.9	2.10E-13
GO:0016072	rRNA metabolic process	63	38	12.29	9.20E-13
GO:0055114	oxidation-reduction process	1002	277	195.53	1.80E-12
GO:0034470	ncRNA processing	99	46	19.32	8.00E-10
GO:0005975	carbohydrate metabolic process	379	121	73.96	1.60E-09
GO:0044085	cellular component biogenesis	133	55	25.95	3.80E-09
GO:0005976	polysaccharide metabolic process	107	46	20.88	1.70E-08
GO:0034660	ncRNA metabolic process	147	56	28.69	8.70E-08
GO:0000272	polysaccharide catabolic process	95	41	18.54	9.20E-08
GO:0016052	carbohydrate catabolic process	152	55	29.66	8.10E-07
GO:0044710	single-organism metabolic process	1658	388	323.54	1.10E-06
GO:0030243	cellulose metabolic process	20	12	3.9	7.60E-05
GO:0030245	cellulose catabolic process	20	12	3.9	7.60E-05
GO:0051275	beta-glucan catabolic process	20	12	3.9	7.60E-05
GO:0009251	glucan catabolic process	23	13	4.49	9.00E-05
GO:0044042	glucan metabolic process	29	15	5.66	0.0001
GO:0051273	beta-glucan metabolic process	21	12	4.1	0.00015
GO:0006073	cellular glucan metabolic process	27	14	5.27	0.00017
GO:0006631	fatty acid metabolic process	41	18	8	0.0003
GO:0044275	cellular carbohydrate catabolic process	35	16	6.83	0.00037
GO:0006633	fatty acid biosynthetic process	32	15	6.24	0.00041
GO:0071840	cellular component organization or bioge...	304	83	59.32	0.00043
GO:0006396	RNA processing	193	57	37.66	0.00043
GO:0044247	cellular polysaccharide catabolic process	29	14	5.66	0.00044
GO:0008643	carbohydrate transport	68	25	13.27	0.00064
GO:0010410	hemicellulose metabolic process	24	12	4.68	0.00075
GO:0044262	cellular carbohydrate metabolic process	69	25	13.46	0.00082
GO:0044036	cell wall macromolecule metabolic process	44	18	8.59	0.00086
**CC**					
GO:0005730	nucleolus	50	36	8.95	3.70E-17
GO:0005576	extracellular region	136	59	24.34	1.40E-12
GO:0031981	nuclear lumen	104	37	18.61	9.30E-06
GO:0043233	organelle lumen	135	43	24.16	4.20E-05
GO:0070013	intracellular organelle lumen	134	42	23.98	7.90E-05
GO:0031974	membrane-enclosed lumen	144	43	25.77	0.00022
GO:0031224	intrinsic to membrane	943	200	168.78	0.00103
